# PIRT-Seq: a high-resolution whole-genome assay to identify protein-coding genes

**DOI:** 10.1093/nar/gkaf774

**Published:** 2025-08-13

**Authors:** Emily C A Goodall, Freya Hodges, Weine Kok, Budi Permana, Thom Cuddihy, Zihao Yang, Nicole Kahler, Kenneth Shires, Karthik Pullela, Von Vergel L Torres, Jessica L Rooke, Antoine Delhaye, Jean-François Collet, Jack A Bryant, Brian M Forde, Matthew R Hemm, Ian R Henderson

**Affiliations:** Institute for Molecular Bioscience, University of Queensland, Brisbane 4072, Australia; Environment and Sustainability Institute & Centre for Ecology and Conservation, University of Exeter, Penryn, TR10 9FE, United Kingdom; Institute for Molecular Bioscience, University of Queensland, Brisbane 4072, Australia; Institute for Molecular Bioscience, University of Queensland, Brisbane 4072, Australia; Institute for Molecular Bioscience, University of Queensland, Brisbane 4072, Australia; Institute for Molecular Bioscience, University of Queensland, Brisbane 4072, Australia; Institute for Molecular Bioscience, University of Queensland, Brisbane 4072, Australia; Department of Biological Sciences, Towson University, Towson, 21252-0001 United States; Department of Biological Sciences, Towson University, Towson, 21252-0001 United States; Institute for Molecular Bioscience, University of Queensland, Brisbane 4072, Australia; Institute for Molecular Bioscience, University of Queensland, Brisbane 4072, Australia; Institute for Molecular Bioscience, University of Queensland, Brisbane 4072, Australia; Institut de Duve, UC Louvain, Brussels 1200, Belgium; Institut de Duve, UC Louvain, Brussels 1200, Belgium; School of Life Sciences, University of Nottingham, Nottingham, NG7 2UH, United Kingdom; Institute for Molecular Bioscience, University of Queensland, Brisbane 4072, Australia; Centre for Clinical Research, University of Queensland, Brisbane 4072, Australia; Department of Biological Sciences, Towson University, Towson, 21252-0001 United States; Institute for Molecular Bioscience, University of Queensland, Brisbane 4072, Australia

## Abstract

The advent of high-density mutagenesis and data-mining studies suggest the existence of further coding potential within bacterial genomes. Small or overlapping genes are prevalent across all domains of life but are often overlooked for annotation and function because of challenges in their detection. To overcome limitations in existing protein detection methods, we applied a genetics-based approach. We combined transposon insertion sequencing using a dual-selection transposon with a translation reporter to identify translated open reading frames throughout the genome at scale but independent of genome annotation. We applied our method to the well-characterised species *Escherichia coli*. This method revealed over 200 putative novel protein coding sequences (CDS). These are mostly short CDSs (<50 amino acids) and include proteins that are highly conserved and neighbour functionally important genes. Using chromosomal tags, we validated the expression of selected CDSs. We present this method (Protein Identification through Reporter Transposon-Sequencing: PIRT-Seq) as a complementary method to whole cell proteomics and ribosome trapping for condition-dependent identification of protein CDSs, and as a high-throughput method for testing conditional gene expression. We anticipate this technique will be a starting point for future high-throughput genetics investigations to determine the existence of unannotated genes in multiple bacterial species.

## Graphical Abstract

**Graphical Abstract:**
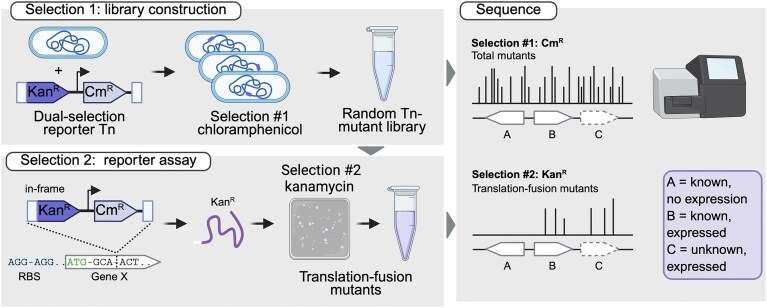

## Introduction

The last two decades have seen a substantial increase in sequenced bacterial genomes, coupled with an increase in the associated pan-genome of any given organism. However, in recent years, there has been a growing body of work to suggest that our annotation of bacterial genomes is incomplete. First, smaller proteins have been historically overlooked in all domains of life [1–4]. Early definitions of a gene or coding sequence applied a size cut-off for annotation of a gene, ranging from 50 to 100 codons. However, several translated open reading frames (ORFs) as small as two or three codons have been identified in *Escherichia coli* and other species [5–7]. A small protein, as defined by Storz and colleagues, is directly translated from an ORF (as opposed to processed from a larger protein), and composed of fewer than 50 amino acids [4]. Small proteins (also referred to as ‘short ORF-encoded proteins’ (SEPs), ‘microproteins’ or ‘sproteins’) have been identified across the bacterial kingdom (reviewed extensively elsewhere [8–10]), with roles in stress response (e.g. Prli42, TisB [11–13]), cell division (e.g. MciZ, SidA, Blr [14–17]), metabolism (e.g. SgrT, MgtS, MntS [18, 19]), respiration (e.g. CcoM, CydX [20, 21]), signal transduction (e.g. Sda, MgrB [22, 23]), modulating antibiotic resistance (e.g. Blr, AcrZ, Prli53 [6, 24]), defence and toxin/antitoxin systems (PepA1, TisB, Fst [12]), or mediating host-interaction (e.g. rio3 [25]) and survival inside host cells (KdpF [26]). Given their functional importance, a number of studies have sought to identify these previously overlooked small proteins at scale [27], but this has not been without its challenges. Small proteins are harder to predict bioinformatically as the short sequence length makes it difficult to distinguish true coding sequences from random in-frame sequences between a start and stop codons [28]. Furthermore, they can be difficult to predict through sequence conservation analysis as they can be genus or lineage restricted [29].

Another class of overlooked genes are nested genes. Nested genes are defined as those encoded within another gene [30, 31]. They can be encoded within the same orientation as the primary coding sequence or anti-sense to the primary transcript. A subset of nested genes are isoforms [32], which are encoded from an alternate start codon within the primary transcript, such as *cheA*(L) and *cheA*(S) in *E. coli* [33], or the three isoforms of translation initiation factor 2 encoded by *infB* [34, 35]. Nested genes are prevalent in viruses and eukaryotes due to their compact genomes or coding complexity, respectively, but considered rare anomalies in bacterial genomes [36]. As such, many automated bacterial genome annotation tools discard putative coding sequences that are within larger genes. However, well-documented examples of (non-isoform) nested genes include the *Shigella* enterotoxin (ShET) 1 gene encoded antisense to the Pic autotransporter [37], which are both virulence factors of diarrhoeagenic *E. coli* isolates, and *comS* encoded in the same sense but out of frame (OOF) to *srfA* in *Bacillus subtilis*. Interestingly, *comS* and *srfA* have complementary but independent functions in natural competency (*comS*) and surfactin synthesis and secretion (*srfA*), respectively [38]. More recent discoveries of overlapping genes (*olg1* and *olg2*, respectively) as large as 957 and 1728 nucleotides have been demonstrated in *Pseudomonas aeruginosa* [39], suggesting overlapping protein coding is not limited to just small proteins.

Due to the limitations outlined above, high-throughput methods to identify protein coding sequences are needed to fully elucidate the genetics and biology of a given organism. Methods that trap mRNA-bound ribosomes (Ribo-seq) have been developed to identify translated mRNA and therefore protein coding sequences [32, 40, 41]. Although these studies highlight the remarkable extent to which small proteins have been overlooked, the data can however pose challenges for interpretation [42]. Ribo-Seq methods have been further optimised through the use of antibiotics that trap translating ribosomes at specific positions along translated mRNA [43]; one example termed ‘Ribo-Ret’ uses retapamulin, which traps initiating ribosomes resulting in enrichment of ribosomes localised near the initiating start codon. Such adaptations improve the resolution of the output data, but pose other challenges such as differentiating true coding sequence from ‘pervasive translation’ [44]. Small proteins are also challenging to detect via mass spectrometry (discussed in detail by Ahrens *et al.* [45]). They might be transiently expressed, expressed only under specific conditions [46], low in abundance, have few sites for trypsin digestion, or highly hydrophobic membrane-associated proteins. Taken together, it is clear that complementary methods are needed if we are to fully realise the coding potential of any given organism.

Translation reporters are one tool for identification of protein coding sequences. Such assays involve the fusion of the *C*-terminus of the target protein to the *N*-terminus of an enzyme or protein with a measurable read out, such as *β*-galactosidase, alkaline phosphatase, or a fluorescent protein. Most translation reporter assays have been applied to defined target sequences, often in isolation. However, an early transposon-based random mutagenesis approach introduced the *phoA* gene, encoding alkaline phosphatase, into a Tn*5* transposon derivative ‘Tn*phoA*’. The transposon was designed such that the alkaline phosphatase, which is only functional in the periplasm, lacked its signal peptide sequence and therefore would only be functional if fused to sequences that promote its export [47], thereby identifying secreted proteins and periplasmically located portions of integral membrane proteins. This approach pre-dated the high-throughput amplicon-sequencing technology available today but demonstrates the utility of a transposon-based reporter system. Here, we combined a mini-Tn*5* translation reporter with ultra-dense transposon mutagenesis and sequencing to query the *E. coli* K-12 genome for new protein coding sequences independently of existing genome annotation at scale. By using this approach, we identified previously uncharacterised protein coding genes. We also document their conservation among >200 000 *E. coli* genomes. The capacity to discern an organism's coding potential in a single experiment promises to be a powerful strategy for unveiling genotype–phenotype relationships.

## Materials and methods

### Bacterial strains and culture conditions

*E. coli* K-12 strain BW25113 (*rrnB*_T14_ Δ*lacZ*_WJ16_*hsdR514* Δ*araBAD*_AH33_ Δ*rhaBAD*_LD78_) was routinely used. Cell cultures were grown overnight in LB broth (Tryptone 10 g/L, Yeast extract 5g/L, Sodium Chloride 10g/L) at 37°C supplemented with 100 μg/ml carbenicillin (Sigma) for plasmid selection and 35 μg/ml chloramphenicol (Merck) or 25 μg/ml kanamycin (Sigma) for transposon selection.

### Cloning

The transposon sequence was constructed by stitch polymerase chain reaction (PCR) using the commercially available EZ-Tn*5* Kan^R^ transposon (Lucigen), and a previously published Cm^R^ mini-Tn*5* transposon [49] as a template and cloned into the pUC19 vector. The oligonucleotides used in this study are listed in Supplementary Table S3. The transposon was inserted between the *Hin*dIII and *Bam*HI restriction sites, resulting in the plasmid ‘pUC19-KC-Tn’, which contains the dual selection transposon inserted in-frame in the *lacZ*α gene. Mutations of the *lacZ*α start codon were introduced via site-directed mutagenesis. Briefly, the plasmid was amplified by PCR using phusion polymerase (NEB) and overlapping forward and reverse primers that contain the desired mutation. The vector template was digested by DpnI (NEB) following the supplier’s instructions. Newly amplified vector was transformed into competent DH5alpha cells (NEB), and correct mutations were confirmed by Sanger sequencing before transferring to *E. coli* strain BW25113.

### Construction of a transposon library

To make the transposome (transposon coupled with transposase), the pUC19-KC-Tn plasmid was linearised by restriction digest using *Nde*I (New England Biolabs) following the supplier’s instructions. The mini-Tn*5* transposon was amplified by PCR using primers with a phosphorylated 5′ end and containing the inverted repeat mosaic ends for efficient Tn5 transposase-mediated transposition. The transposon was amplified using the phusion polymerase (NEB) and the linearised plasmid as a template with the following conditions: 30 s 98°C; (10 s 98°C, 20 s 60°C, 1 min 72°C) x30; 5 min 72°C; 4°C hold. The PCR product was purified via gel extraction (Qiagen) following the supplier’s instructions. The Transposon was eluted in TE buffer (pH 8.0; ThermoFisher Scientific), quantified by Qubit dsDNA HS Assay kit (ThermoFisher Scientific) and adjusted to a final concentration of 100 ng/μl. To prepare the transposome, a ratio of 1:2:1 of 100 ng/μl transposon DNA in TE buffer, EZ-Tn5 Transposase (TNP92110; Gene Target Solutions) and 100% glycerol were incubated at RT for 1 h then frozen at −20°C for storage. The transposome was introduced into *E. coli* K-12 strain BW25113 by electrotransformation. Briefly, cells were grown to mid-exponential phase in 800 ml LB at 37°C with aeration, harvested by centrifugation at 2000 xg for 10 min at RT and washed 4x in 10% glycerol at the same volume. The final cell pellet was resuspended in 1 ml of 10% glycerol. Aliquots of 100 μl cells were incubated with 1 μl transposome before shocking at 25 kV. For recovery, 1 ml LB was added per reaction, and cells were incubated a 37°C with shaking for 2 h. Successful transformants were selected on LB agar plates supplemented with 35 μg/ml chloramphenicol and incubated overnight at 37°C for 18 h. Colonies were scraped from plates to form the transposon library of pooled mutants and resuspended in 30% glycerol in LB for storage at −80°C. For selection of kanamycin resistant mutants, 25 μg/ml of kanamycin was identified as the minimum concentration of kanamycin that inhibits growth of *E. coli* BW25113 on supplemented LB agar plates (Supplementary Fig. S4A) and was therefore selected as the concentration for selection of kanamycin-resistant isolates. For identification of mutants expressing the translation reporter, the transposon library was selected on LB agar plates supplemented with 25 μg/ml kanamycin, to achieve a density of ∼2000 colonies per plate. A total of ∼1 × 10^7^ CFUs, of the input library were screened in total (per replicate), ensuring sufficient coverage of the library. Colonies were scraped from plates, resuspended in 30% glycerol-LB, pooled, and stored at −80°C.

### Transposon sequencing

Genomic DNA (gDNA) was extracted, fragmented by ultrasonication to an average size of ∼250 bp and prepared for sequencing using the NEB Next Ultra I kit (E7370L, NEB), with some modifications. Following end repair, adapter ligation, and a size-selection purification step with SPRI beads (Beckman Coulter) following the kit instructions, a PCR step was introduced to enrich for Transposon-gDNA junctions, using a forward primer specific for the transposon (Kan^R^ gene; Supplementary Fig. S2) and a reverse primer specific for the NEB adapter. The following were mixed: 15 μl DNA fragments in nuclease free water, 25 μl Q5 polymerase, 2.5 μl 10 μM each primer, and 5 μl nuclease free water to a final volume of 50 μl, and amplified in a thermocycler with the profile: 3 min 98°C; (15 s 98°C, 30 s 65°C, 30 s 72°C) x10; 1 min 72°C; hold 4°C. The sample was purified using SPRI beads at a ratio of 0.9:1 beads to sample. A second PCR step then prepared the transposon-gDNA junctions for sequencing using custom forward primers specific for the transposon, which incorporated a barcode for sample identification as well as staggering the start of the transposon sequence during sequencing. The reverse primers, NEBNext Multiplex Oligos for Illumina (E7335, NEB), have homology to the adapter. The purpose of this step is to introduce barcode sequences and further adapters to enable fragment binding to the sequencing flow cell; the fragments were prepared as before with the following conditions: 3 min 98°C; (15 s 98°C, 30 s 65°C, 30 s 72°C) x20; 1 min 72°C; hold 4°C. The final product was purified again using SPRI beads and then quantified by qPCR following the kit instructions (KAPA Library Quantification Kit, Roche). Fragments were sequenced using an Illumina MiSeq using v3 150 cycle cartridges (MS-102–3001, Illumina), with 5% PhiX loading control (Illumina).

### Transposon data analysis

Sequencing data were processed using a previously described approach [50]. Briefly, FASTQ data were first demultiplexed by the internal barcodes (introduced by the forward primer of PCR2) using the FASTX-Toolkit (http://hannonlab.cshl.edu/fastx_toolkit/index.html). Samples were then checked for the transposon sequence in two steps allowing for a total of 4 bp mismatches. Following identification of transposon-containing reads, the transposon sequence was trimmed and the remaining read mapped using bwa mem (https://bio-bwa.sourceforge.net/bwa.shtml#13), to the *E. coli* BW25113 reference genome (available from the NCBI database, accession CP009273.1, unless otherwise stated). The first nucleotide of a given read was used as the transposon insertion site to generate insertion plot files, which were viewed in Artemis [51]. We used bbcountunique.sh of BBTools for sub-sampling of FASTQ data (https://sourceforge.net/projects/bbmap/). Insertion data were cross-referenced with annotated genome features using intersectBed [52]. Where an intergenic, or intragenic but out-of-frame with the annotated gene, translation-reporter insertion was identified, the reading frame (region between two stop codons) was reviewed for any of seven putative start codons (AUG, GUG, UUG, CUG, AUA, AUU, AUC) in preferential order. To be conservative, where >1 start codon was identified, the furthest upstream was used. The identification of translation within a reading frame and presence of a start codon resulted in the final dataset of 215 putative CDSs, the sequences of which are listed in Supplementary Table S2.

### Construction of SPA-tagged strains

A sequential peptide affinity (SPA) tag (containing the calmodulin binding peptide (CBP) and 3 × FLAG sequences separated by a TEV Protease cleavage site) was introduced in-frame into the chromosome immediately upstream of the target CDS stop codon, resulting in a *C*-terminal tag [53, 54]; a tag routinely used for the identification of small proteins [41, 54, 55]. The SPA tag was synthesised by GenScript and stitched by PCR [56], with a kanamycin resistance cassette flanked by two FRT sites amplified from the *clsA* Keio mutant [57]. The SPA-Kan^R^ fragment was subcloned into vector pJET1.2 (ThermoFisher Scientific), and the vector used as a template for PCR amplification of the SPA-tag and kanamycin resistance cassette using primers with 50 bp flanking regions homologous to the intended chromosomal insertion site. PCR profile: 98°C 1 min; (98°C 10 s, 62°C 10 s, 72°C 2 min)x34; 72°C 2 min. Gel purified linear fragments were electroporated into BW25114/pKD46 via the Datsenko-Wanner method of chromosomal mutation [48]. All mutants were verified first by PCR (Supplementary Fig. S8) and Sanger sequencing.

### Western blot analysis

Immunoblot assays to determine small protein levels were conducted as described previously with minor modification [54]. In brief, overnight cultures were sub-cultured 1:500 into LB and grown at 37°C with aeration to either mid-exponential phase (OD_600_ = 0.3–0.6) or stationary phase (OD_600_ = 4.0–5.0). Cells were harvested by centrifugation and resuspended in 50 mM sodium phosphate buffer (pH 8). Re-suspended whole cells were mixed with 4 × sample buffer (1 × stacking buffer, 2% SDS, 0.025 mg bromophenol blue, 52% glycerol), heated at 95°C for 10 min and centrifuged for 10 min. Samples were separated on Novex 16% Tricine gels (ThermoFisher Scientific) and transferred to nitrocellulose membranes (ThermoFisher Scientific). The membranes were blocked with 2% milk and then probed with anti-FLAG M2-HRP monoclonal antibody (A8592, MilliporeSigma) in PBS-T. Signals were visualised using SuperSignal West Dura Extended Duration Substrate (ThermoFisher Scientific). Equal loading of lanes in each gel was confirmed by examining the relative intensity of background bands in the test samples as compared to the untagged, wild-type control samples.

### Protein structure predictions

Protein secondary structure were predicted using PSIPRED (4.0) with MEMSAT-SVM for TM-domain prediction, using the default settings (psipred (v4.1): dataset uniref90, hhdb uniclust30 (v2018_08), psiblast (v2.2.26), hhblits (v2.0.16); memsatsvm: uniref90, psiblast (v2.2.26), run_memsat-svm (v1.3)) [58, 59]. Protein structures were predicted using ColabFold (v1.5.5): AlphaFold2 using MMseqs2 and the default settings [60].

### Conservation analysis within *E. coli*

To investigate the prevalence and distribution of short open reading frames (sORFs) among *E. coli* strains, we screened a collection of globally distributed *E. coli* genomes. We downloaded 227 873 *E. coli* genome assemblies, along with all available metadata, from Enterobase (accessed on 14 March 2024) [61]. The presence of CDSs was determined using tBLASTn (version 2.12.0+). The tBLASTn analysis was conducted with default settings, but adjustments were made to account for the short length of the query sequences: -matrix PAM30 and -word_size 5.

### Identification of small protein homologues outside *E. coli*

Potential homologues of the *E. coli* small proteins were identified similar to as described [62]. tblastn searches were conducted of bacterial species genomes using the National Center for Biotechnology Information (NCBI) microbial database. Only species that were labelled as ‘complete genomes’ by NCBI were screened. Each sORF was searched individually. All *Escherichia* (taxid:561) and *Shigella* (taxid:620) genomes were excluded from these searches. Homologues were searched in both ‘Representative genomes only’ and ‘All genomes – Complete genomes’ Organism options. Other settings are as follows: Max target sequences of 1000, Expect threshold of 1000 or 10 000, Word Size of 2, BLOSUM62 Matrix, Gap Costs of ‘Existence: 11 Extension: 1’ and ‘Conditional compositional score matrix adjustment’. Finally, the low complexity regions filter was turned off in all cases. Unannotated potential homologues were identified through manual analysis of the NCBI Gene database. Potential start codons and stop codons were identified using the Expasy Translate tool to analyse the genomic sequence (web.expasy.org/translate/). Multiple sequence alignments (MSAs) were created using the ClustalW program (www.genome.jp/tools-bin/clustalw).

## Results

### Construction of a reporter-transposon library

**Figure 1.**
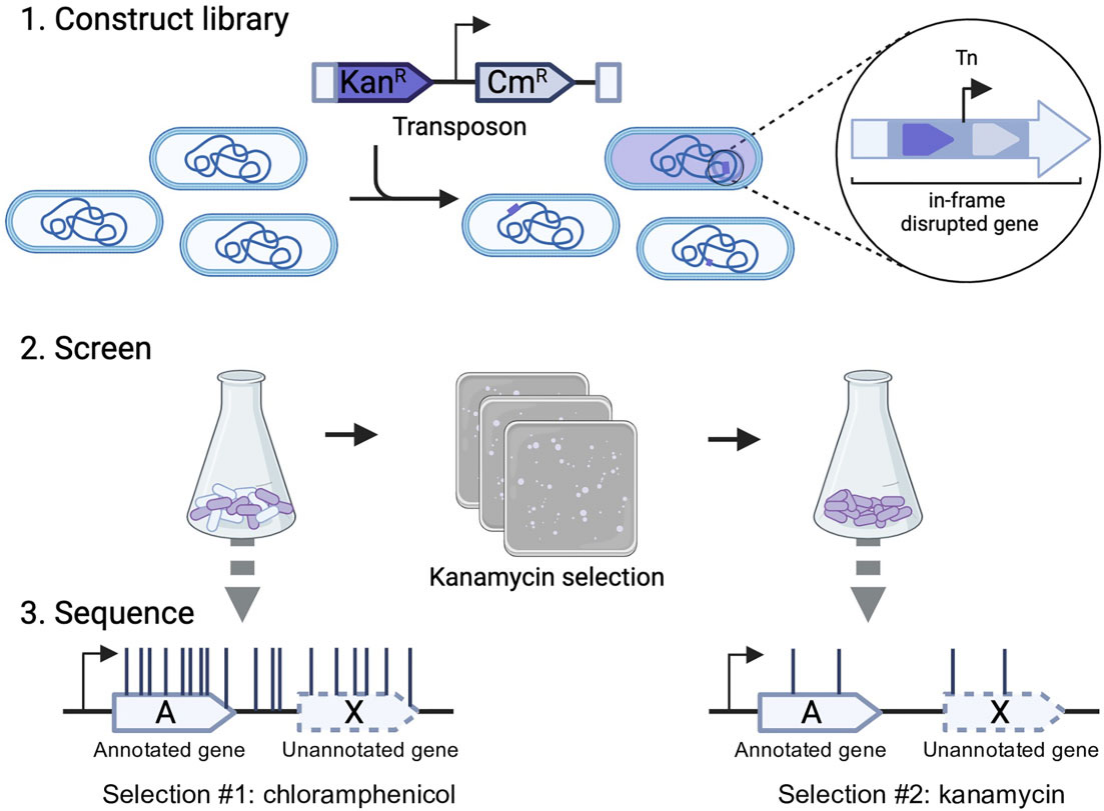
Method overview. [1] Construction of a transposon mutant library via introduction of a transposon with a dual selection mechanism. Successful transformants are isolated via selection on LB agar supplemented with chloramphenicol. [2] Screening of the transposon library on LB agar supplemented with kanamycin selects for mutants that contain a transposon inserted in-frame within an expressed protein coding sequence. [3] Sequencing of the input and output transposon mutant pools reveals translation-fusion mutants that resulted in the expression of the kanamycin resistance cassette, and therefore identifies protein coding sequences. Known, annotated genes (gene A) are indicated by a closed line arrow, while unannotated genes (gene X) are represented with a dashed line arrow, to demonstrate how the insertion data can reveal new genes.

In our previous research [49], the resolution afforded from ultra-dense transposon mutagenesis highlighted the presence of newly annotated small genes that contribute to cell fitness. We hypothesised that there might be additional as yet unannotated coding sequences in the *E. coli* genome, and that a transposon mutagenesis approach could be leveraged for their detection. A dual-selection reporter transposon system was constructed by introducing a kanamycin resistance gene upstream of a chloramphenicol resistance cassette in a mini-Tn*5* transposon. However, the transposon was designed such that the aminoglycoside-3′-phospotransferase (*aph*) gene that confers kanamycin resistance was introduced immediately after the mini-Tn*5* inverted repeat without a promoter, ribosome binding site (RBS) or start codon (Fig. 1). Expression of the *aph* gene is dependent upon in-frame insertion of the transposon into an actively translated gene. Before library construction, we first confirmed that the intact antibiotic selection cassettes did not confer any cross resistance (Supplementary Fig. S1A). We next verified that the *aph* gene could confer kanamycin resistance with an N-terminal fusion of the transposon inverted repeat at the 5′ end of the *aph* gene. The transposon was cloned in-frame into *lacZ*α in a pUC19 vector such that expression of *aph* was dependent upon Plac and the start codon of *lacZ*α. The *lacZ*α-Tn fusion was sufficient to confer kanamycin resistance, and this resistance was lost if the *lacZ*α start codon is mutated from ATG to AGG (Supplementary Fig. S1B and C). Having confirmed the functionality of the transposon, we introduced the transposon via transposition into the *E. coli* K-12 strain BW25113 genome at random aiming for one transposon insertion event per cell. We used the mini-Tn*5* system because it can achieve near-saturating mutagenesis [49], enabling maximum genomic coverage of transposon mutants in the input pool. Transposon mutants were selected on LB agar plates supplemented with chloramphenicol and were pooled to form the library. The transposon-gDNA junctions were sequenced to identify the transposon insertion site. We designed our sequencing primers to sequence from the kanamycin resistance gene into the gDNA upstream to directly identify CDS translation fusion sites (Supplementary Fig. S2). We sequenced two aliquots of the library and obtained 4.2 M and 2.7 M sequencing reads per replicate respectively. Comparison of the insertion density per gene between technical replicates showed sequencing the transposon junction was reproducible between replicates with a Pearson correlation coefficient of 0.99 (Supplementary Fig. S3A); therefore, we pooled the replicate data resulting in a total of ∼6.9 M mapped reads. Sub-sampling of our transposon junction sequences confirmed we had obtained sufficient sequencing for full coverage of our library (Supplementary Fig. S3B). We identified 185 709 and 184 405 unique insertions for each orientation of the transposon respectively representing a total of 370 114 translational reporter mutants. When we further delineated the data by reading frame, there was an equivalent number of mutants for each frame (Supplementary Fig. S3C), distributed throughout the genome (Supplementary Fig. S3D).

### Identification of putative new genes

**Figure 2.**
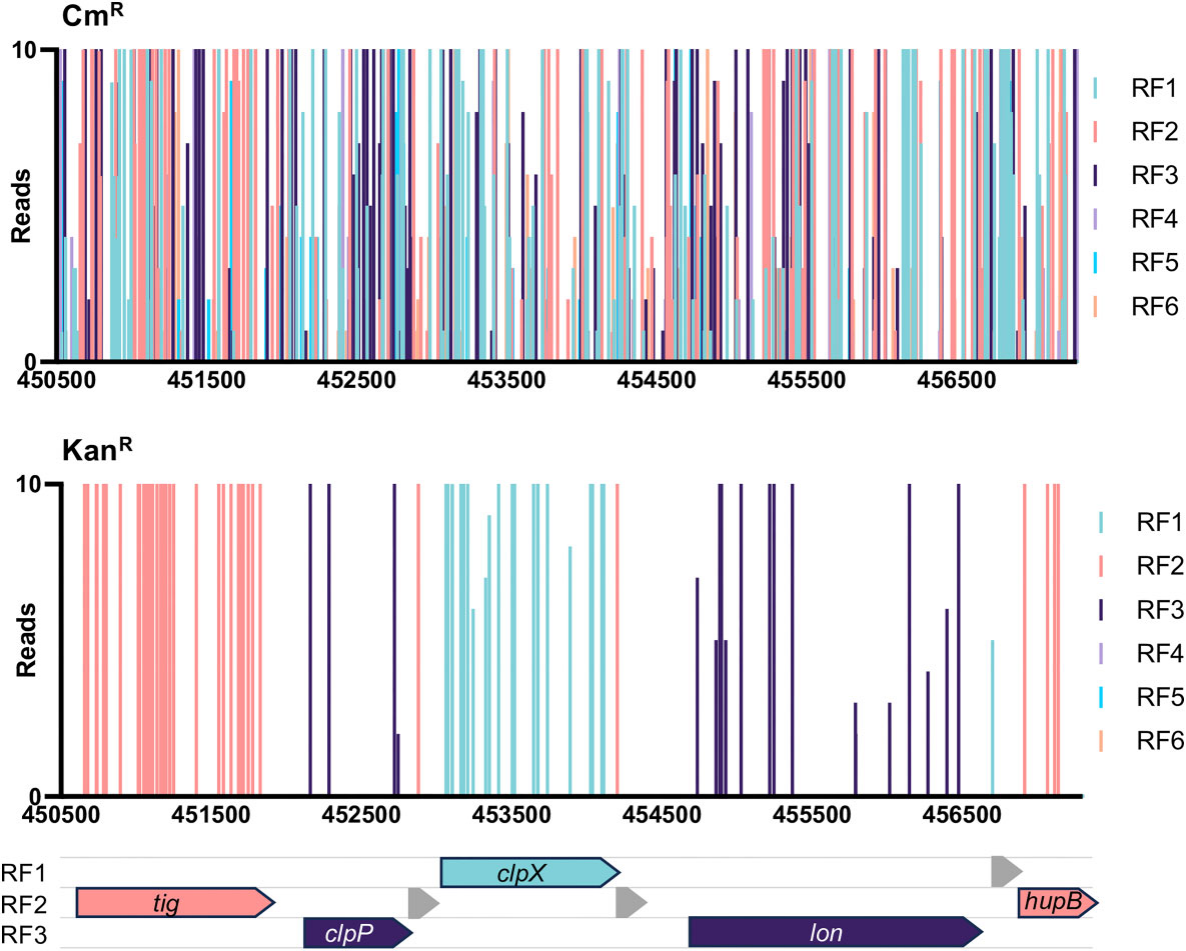
The bp resolution of the translation reporter reveals the reading frame of protein coding sequences. The initial construction of the transposon library was selected on agar plates supplemented with chloramphenicol (top panel). The vertical bars represent both the frequency and position of identified transposon insertion sites. The data are coloured according to the reading frame (RF) at the site of insertion. A secondary selection on agar plates supplemented with kanamycin selected for mutants where the transposon is inserted in frame of a protein coding sequence. The data are coloured according to the reading frame at the point of insertion and are consistent with the annotated genes (bottom panel). Annotated genes are indicated by filled arrows with gene names while putative new protein coding genes are indicated by grey arrows.

To identify expressed protein coding sequences, we repeated selection of the library on agar plates supplemented with an inhibitory concentration of kanamycin, with the expectation that kanamycin resistance will only be conferred if the transposon has inserted in frame into an expressed protein coding sequence. For selection of kanamycin resistant mutants (and therefore translation-fusion mutants), 25 μg/ml of kanamycin was identified as the minimum concentration of kanamycin that inhibits growth of the parent strain *E. coli* BW25113 on supplemented LB agar plates at this cell density while selecting for transposon mutants (Supplementary Fig. S4A and B). We chose to screen the library at the minimum inhibitory concentration to reduce the selection stress and maximise the number of translation-fusion mutants recovered. The library was screened twice, on separate days. We sequenced the transposon-gDNA junctions as before and recovered > 150 000 reads per sample, which mapped to ∼8 000 unique insertion sites per replicate. The biological replicates of mutants selected on kanamycin plates had a strong Pearson correlation coefficient (*r* = 0.81) between identified insertion sites (Supplementary Fig. S5A). The number of recovered unique insertions was lower than the number of fusion mutants we expected to recover. This may be a result of disrupted mRNA stability when endogenous CDSs are disrupted mid-sequence: indeed, review of the relative position of insertion sites found they were predominantly within the extreme 5′ or 3′ of each CDS, regardless of gene essentiality (Supplementary Fig. S5B–D). To account for the possibility that individual mutants might grow if they acquire spontaneous kanamycin resistance, we filtered the data to include hits that were identified in both independent screens, resulting in a final number of 6219 transposon insertion sites. We then reviewed the functionality of the screen: we split the insertion data by reading frame and confirmed the bp level of resolution for identification of translated reading frames. For example, within the *tig-clpP-clpX-lon-hupB* operon the transposon insertions were each consistent with the appropriate reading frame for translation fusion with the kan^R^*aph* gene (Fig. 2), demonstrating the functionality of the screen. Moreover, this level of resolution can reveal putative CDSs that do not coincide with annotated genes, for example three unannotated putative novel CDSs were identified within the *tig* operon (Fig. 2).

**Figure 3.**
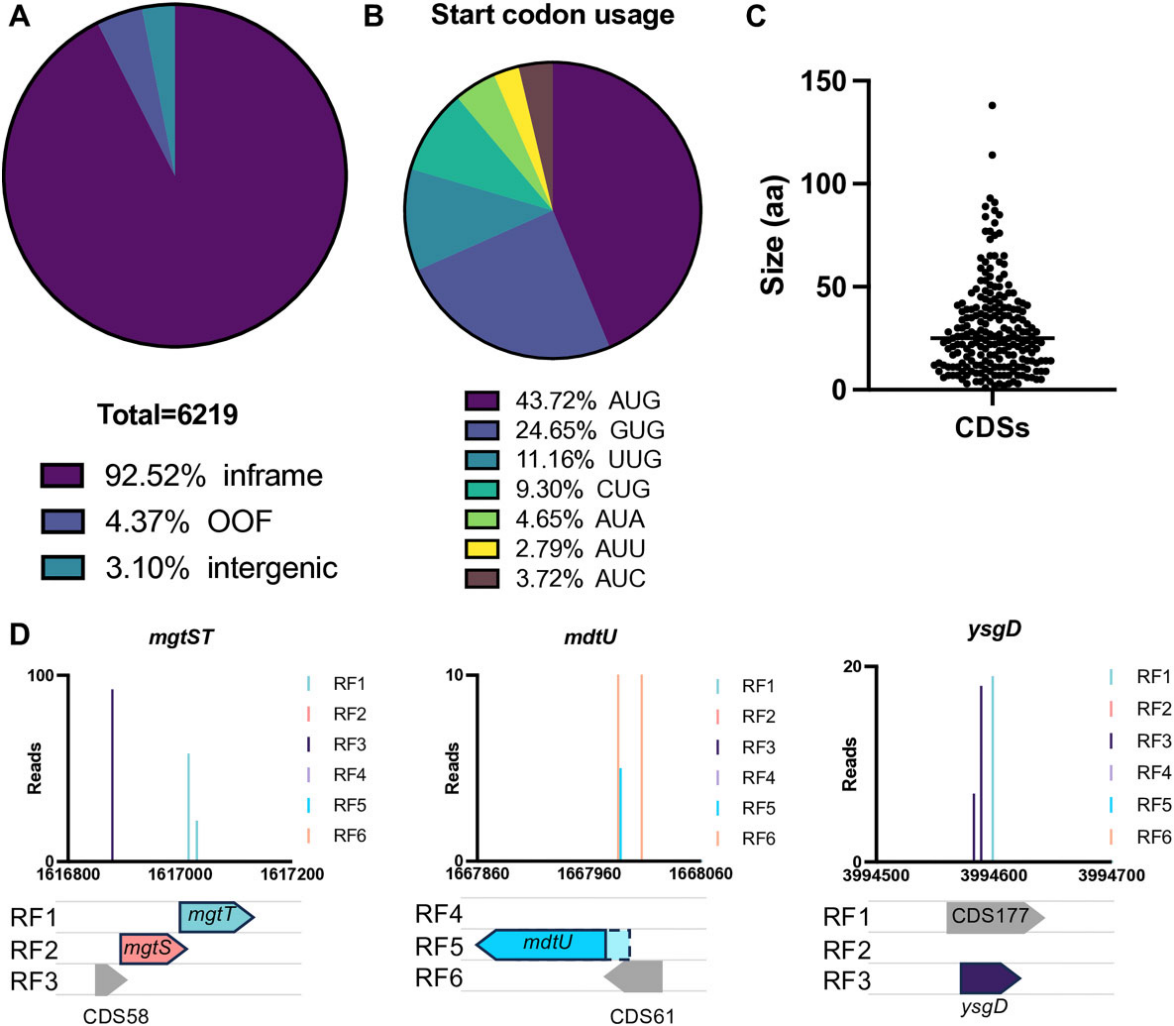
Identification of protein coding sequences. (**A**) The identified transposon-insertion site (and therefore translation fusion junction) was mapped to the reference genome and cross-references with annotation information. Around 92.52% of insertions were within annotated genes and ‘inframe’ consistent with the reading frame of the annotated gene. Around 4.37% of insertions were within annotated genes but in a different reading frame to the annotated gene OOF and 3.10% of insertions were not within annotated protein coding sequences. (**B**) The start codon frequency of 215 putative CDSs, and (**C**) the CDS sizes in amino acids, with the median shown by a horizontal line. (**D**) Small CDSs identified by reporter TIS neighbouring small genes identified by Ribo-Ret[41]. The transposon insertion sites are those identified following selection with kanamycin (representing translation-fusion events) and are coloured according to the reading frame (RF) at the site of translation-fusion. Putative new genes are shown in grey, labelled 'CDS' accordingly.

Of the conservative 6219 insertion sites, 5754 insertions (92.52%) were in-frame within 1139 annotated genes, 272 insertions (4.37%) were within annotated genes but OOF, and 193 insertions (3.10%) were intergenic (between annotated protein coding sequences; Fig. 3A). We used the well curated GenBank sequence (accession CP009273) as a reference for protein coding genes, however, to account for more recently annotated genes we also cross referenced our data against the RefSeq annotation (annotated by the NCBI prokaryotic genome annotation pipeline [PGAP], accession NZ_CP009273, annotated 24-APR-2023). This led to the positive identification of an additional seven genes, five of which (*mgtT*, *pssL*, *ynfU*, *yqgH* and *ysgD*) were identified in a targeted Ribo-Ret screen in *E. coli* K-12 strain MG1655, further validating the utility of our approach to identify small coding sequences [41]. Of the remaining two, *rseD* and *nadS*, *rseD* has been previously validated by chromosomal integration of a *lacZ* translation reporter [63]. While *nadS* (RS26030), although not currently listed in Ecocyc, is annotated in the PGAP reference genome as a hypothetical protein, our data presents the first lab-based evidence for its expression and suggests it is expressed under normal laboratory growth conditions. Overall, the revised total of known genes that were identified in the screen was 1146. We cross-referenced our data against other whole-cell protein-identification datasets, including a proteomic mass-spectrometry dataset and a Ribo-Ret dataset [32, 64], chosen for using a comparable strain (*E. coli* BW25113 or derivative) and growth condition (LB ± supplements; Supplementary Table S1). The largest cohort of proteins were shared by all three methods (Supplementary Fig. S6A); however, there were clearly some method-dependent differences in the proteins identified (discussed extensively elsewhere for proteomics and Ribo-Seq [42, 65, 66]. Transposon mutagenesis screens are inherently limited by their inability to screen regions of the genome that cannot viably be disrupted, such as essential genes. However, we were able to detect some essential genes, such as within the *mur* operon (Supplementary Fig. S6B), where the transposon is in-frame within the extreme 5′ or 3′ end of the protein CDS, the former due to translational read-out from the chloramphenicol resistance marker maintaining downstream expression [49]. Thus, detecting essential proteins is possible with transposon mutagenesis, but requires saturating mutagenesis.

To identify putative new genes, we reviewed the remaining translation-fusion sites that were either within genes but out-of-frame, suggestive of a nested gene, or intergenic and therefore candidate new coding sequences. While the translation-fusion data can reveal ORFs that are translated, it cannot identify which codon is initiating translation. Although the most common start codon in *E. coli* is AUG, one study found 47 (out of 64) codons are capable of initiating translation, albeit at varying strengths [67]. To be conservative we focused on the strongest seven codons that can initiate translation: AUG, GUG, UUG, CUG, AUA, AUU and AUC, which all displayed expression levels >100-fold higher than the control cells [67]. We reviewed both the intergenic and within-gene ‘out-of-frame’ reading frames identified in our translation screen for these start codons and identified a total of 215 unique putative protein coding sequences (S. Table 2); 125 were intragenic ‘nested’ gene candidates while 90 were intergenic. Most annotated genes containing a predicted nested gene had only one internal nested gene (Supplementary Fig. S7), however, there were a handful with multiple predicted nested genes. Although multiple nested genes within a parent gene has been previously documented in *E. coli*, such as within *waaL* which is reported to contain three nested genes [41], another possible explanation for multiple nested candidates within the transposon insertion sequencing (TIS) data is that these arise from ribosome slippage during translation of the primary transcript, as *E. coli* K-12 strains are prone to higher rates of ribosome slippage than other *E. coli* lineages [68], and the genes with ≥ 5 nested genes are some of the most highly expressed proteins [64].

The predominant predicted start codon in the 215 candidate CDSs was AUG (43.72%) followed by GUG (24.65%) and UUG (11.16%) (Fig. 3B). Although the initial aim of this screen was to identify protein coding sequences irrespective of genome annotation, we observed that most of the new CDSs were small proteins < 50 amino acids (aa; median 25 aa, smallest one aa, largest 138 aa; Fig. 3C). We did not apply a lower limit threshold for the size of putative CDS as (1) translation of two codon sequences is known for *E. coli*, although these sequences often represent translation-control mechanisms rather than a functional protein and (2) the calculated sizes are derived from an estimated start codon and therefore might not be accurate. The finding that most putative new CDSs were <50 aa is not entirely surprising and is consistent with the hypothesis that small proteins are prevalent yet have been systematically overlooked during bacterial genome annotation [65]. Surprisingly, comparison of the candidate 215 CDSs with the 68 candidate small proteins identified by Ribo-Seq methods by Weaver *et al.* [41] revealed only nine genes that were identified by both methods. Of note, many of the small proteins identified uniquely by ribosome-profiling appear recalcitrant to transposition, precluding them from our screen. This raises the possibility that small genes have specific regulatory mechanisms that limit their disruption or represent an overlooked class of essential genes. However, many of the small genes detected using both methods have additional neighbouring putative small genes within our data, suggesting organised transcriptional units of small genes. For example, our data is in agreement with the Ribo-Seq data for the identification of small genes *mgtT*, *mdtU* and *ysgD*, but we also detected translation in alternate reading frames neighbouring these genes (Fig. 3D). For *mgtT* and *mdtU*, we identified translation upstream of these genes, which coincided with putative CDSs with a AUG start codon (CDS58 and CDS61 respectively), yet these were not detected by Ribo-Seq methods. Moreover, within *mdtU* we observed translation earlier within the reading frame than the given start codon for *mdtU*, suggesting an alternate start codon further upstream. For *ysgD*, we detected translation both within this reading frame and within an overlapping reading frame, consistent with CDS177, which also has a putative start codon (AUC). Interestingly, Weaver *et al.* [41] observed translation extending beyond the stop codon of *ysgD*, which would be consistent with expression of CDS177, but the absence of an AUG, GUG or UUG start codon filtered CDS177 from their analysis.

### Validation of new genes

**Figure 4.**
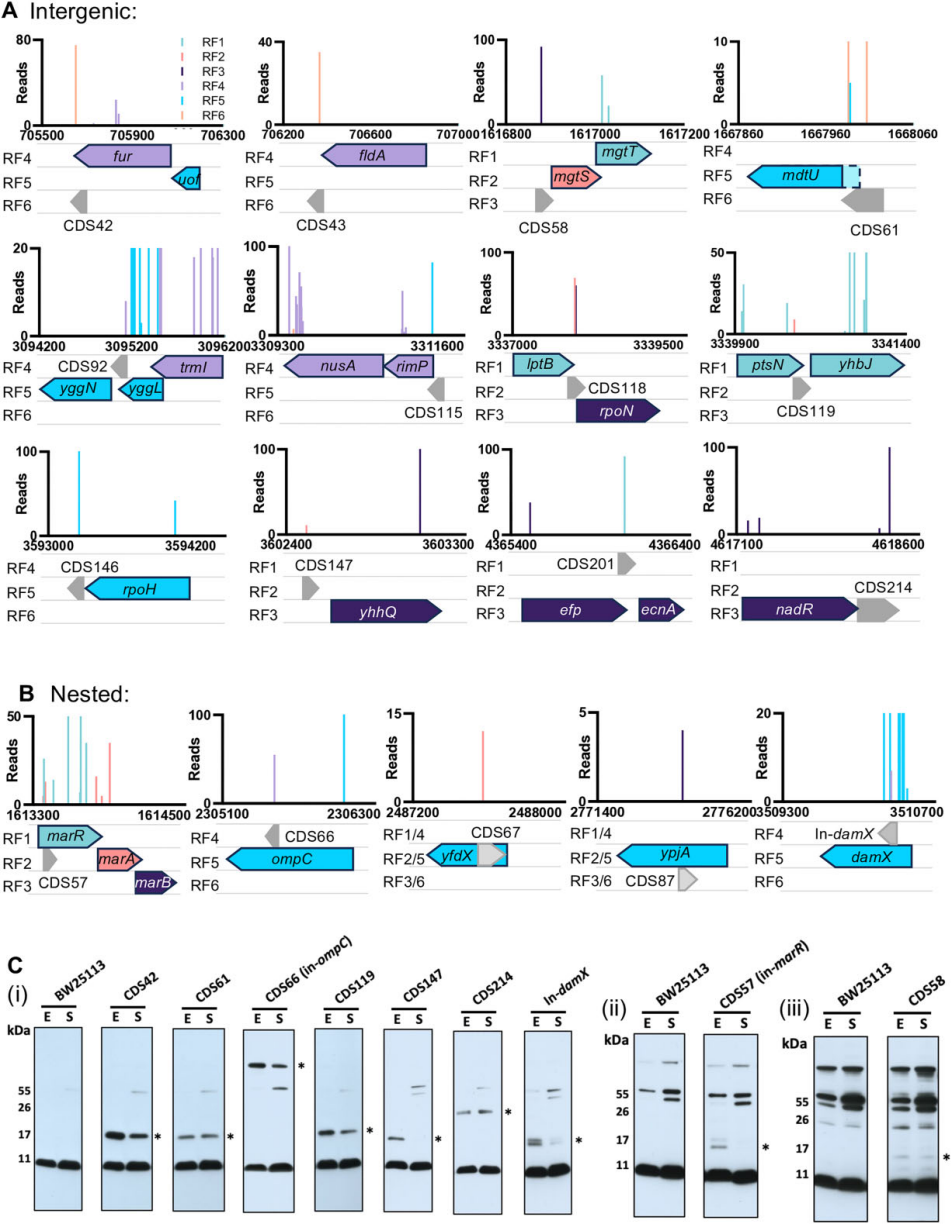
Validation of new proteins. The genetic neighbourhoods of putative (**A**) intergenic and (**B**) nested protein coding sequences (CDS) identified by reporter transposon-insertion sequencing selected for validation. Putative CDSs are shown in grey and labelled 'CDS' accordingly. The insertion data are coloured according to the reading frame (RF) at the site of insertion to highlight the ORF where translation was detected. (**C**) Representative western blots (of 3 repeats): whole cell lysates probed with anti-FLAG antibody, detected proteins are indicated by *. BW = BW25113 control (untagged); E = Exponential phase; S = Stationary phase. Blots are separated into three panels according to the exposure time used for protein detection (i) short (ii) medium (iii) overnight.

To assess the validity of our method, we selected 17 putative new CDSs identified by this method for verification by western blot analysis. We chose candidates with a cross-section of genomic neighbourhoods (start-, mid-, and end-operon), in addition to some intragenic nested genes for proof of principle, and focused on these 17 because of their gene-neighbourhood with some well-known and extensively studied genes (Fig. 4A). Of the 17 putative CDSs, 12 are intergenic and candidate operonic genes, while the remaining five are nested genes but in a different reading frame to the ‘parent’ annotated gene; one of the nested genes (within *damX*) was only identified in one of the kanamycin-selection replicate datasets, but because of its unusual transposon insertion profile we included it for follow-up investigation. Using lambda-red mediated recombination, a dual epitope ‘sequential peptide affinity’ (SPA) tag, comprising a CBP and 3 × FLAG tags, was inserted into the genome immediately upstream of the native stop codon. Constructs were checked by PCR (Supplementary Fig. S8) and confirmed by Sanger sequencing. This approach has been demonstrated previously for the detection of small proteins [54, 69], as the large size of the tag (∼8 kDa) can enable detection of proteins as small as 1 kDa. Strains were grown in LB broth to both exponential and stationary phase, cells were harvested and the whole cell lysates probed with anti-FLAG antibody for the detection of endogenously expressed small proteins labelled with the SPA tag. We detected expression for 6/12 intergenic CDS, and 3/5 of the nested CDSs (Fig. 4B). Small proteins in *E. coli* vary in their detection levels by western blot analysis [69], and there were visible differences in the levels of protein detected here. CDS58, encoded upstream of *mgtST*, had the weakest signal suggesting this protein is not stable or is present at very low levels. We observed that the number of reads for each small protein in the transposon sequencing data did not correlate with the strength of western blot detection, for example, CDS58, which is the faintest on a western blot, had 93 reads, whereas CDS119 and CDS147 were very prominent by western yet only represented by nine and 12 sequencing reads respectively. As such, we did not set a minimum read threshold for CDS detection provided the CDS was detected in two independent kanamycin-selection screens. Importantly, while western blot detection of the SPA tag can validate endogenous translation of a given reading frame, it cannot confirm the start codon for initiating translation. Most of the protein migration patterns were consistent with small proteins, although we noted that the migration of CDS119 was further than expected (suggesting a smaller protein) while the migration of CDS66 was indicative of a larger protein than expected. We cannot make accurate conclusions about protein sizes from migration patterns alone as the amino acid sequence can give rise to aberrant migration such that the observed size is not consistent with the predicted size, as has been observed for Antigen 43 [70], or the protein undergoes some form of posttranslational modification that gives rise to aberrant migration on SDS-PAGE, as has been observed upon lipidation of the *E. coli* CexE protein [71]. However, we note that CDS119 has multiple methionine codons and translation initiated from a start codon further downstream would result in a smaller protein. For CDS66, we confirmed by Sanger sequencing that the correct reading frame was targeted, and the SPA tag is out-of-frame with the *ompC* start codon. Other possible explanations are (1) a homo/heteromultimeric complex that is not reduced by the denaturing conditions when separating the proteins, or (2) translation is initiated from the *ompC* start codon and a + 1 frameshift enables expression of the SPA tag, however the predicted size of this construct (with tag) is ∼34.5 kDa. Further investigation is needed to identify the precise codon initiating translation for these CDSs.

Of the CDSs that we did not detect by western blot analysis, it is possible that they are only transiently expressed, are susceptible to rapid degradation, are expressed at levels below the threshold for western blot detection, or are destabilised by *C*-terminal location of the tag. Indeed, a study validating small proteins identified by Ribo-Seq methods found that some could not be detected by western blot analysis until the addition of a ClpP protease inhibitor, bortezomib [44]. Another possibility is that some of these proteins might be secreted, and therefore missed during our cell harvesting steps prior to lysing the cells for western blot analysis. However, none of the putative CDSs contained any predicted signal sequences [72].

We next predicted the secondary and tertiary structures of the small proteins using PSIPRED and AlphaFold2, including transmembrane (TM) domain prediction using MEMSAT-SVM [58, 60]. CDS42, CDS43, CDS58, CDS61, CDS146, CDS201, and CDS57 were all too short for analysis by PSIPRED (minimum size is 30 residues). All proteins that passed the size threshold for PSIPRED and MEMSAT analysis were predicted to contain a single TM helix, with four of these (CDS118, 119, 147 and 87) predicted to be pore-lining (Supplementary Fig. S9). The AlphaFold2 predictions, generated by the ColabFold pipeline, largely corroborated the secondary structure predictions of PSIPRED, with many helical domains predicted for these small proteins (Supplementary Fig. S10). Although the confidence metrics (pLDDT and pTM) of these predicted structures are low, this likely stems from the limited depth and diversity in the MSAs generated by the ColabFold pipeline as the AlphaFold2 system relies on deep and diverse alignments to generate high-confidence models [60, 73]; however, newly identified proteins may have small sequence families or few homologs in the ColabFoldDB database through which the MSA are generated [60, 73]. Although small proteins are notoriously challenging to classify, the data from three independent structural prediction tools suggest some of these small proteins may be small helical proteins and possibly membrane proteins. Previous studies have reported the prevalence of helical proteins in small protein screens [54], with functions ranging from toxin systems that promote persister cell formation, to accessory subunits of larger membrane protein complexes [74, 75].

### Conservation of new genes

**Figure 5.**
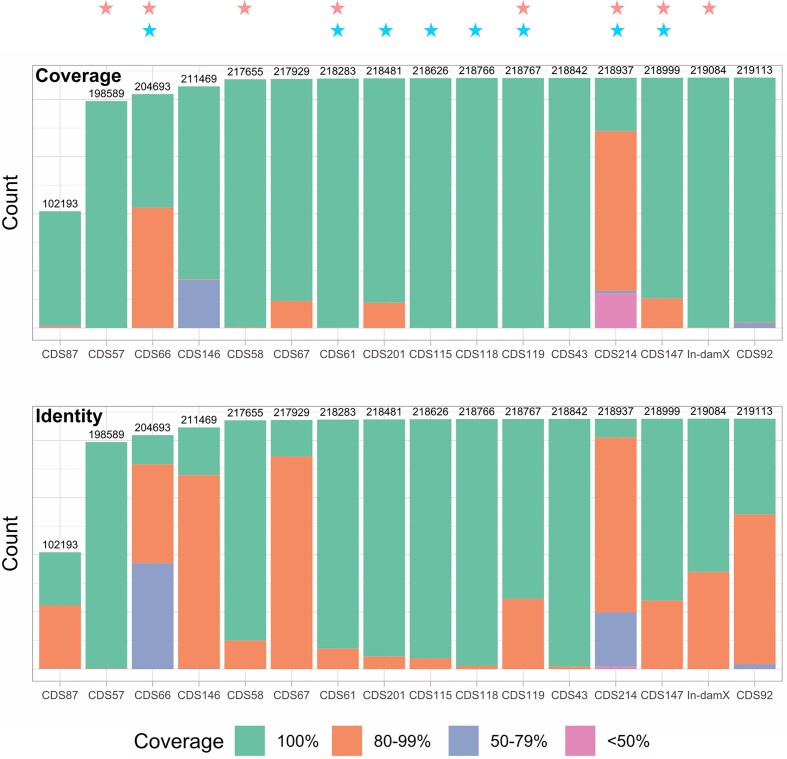
CDS carriage within the 227 873 genome database. tblastn results of CDS homologs within 227 873 *E. coli* genomes, coloured by % coverage and % identity accordingly. CDS validated by western blot analysis are indicated with a pink star (top). CDS with putative homologs outside of *E. coli* are indicated with a blue star (second track).

Finally, we evaluated the conservation of the 17 putative CDS, regardless of western blot detection, to assess whether these coding sequences are conserved. To investigate the prevalence and distribution of the 17 CDSs among *E. coli* strains, we screened a collection of 227 873 globally distributed *E. coli* genomes. Using tblastn analysis, we identified that most of the CDS were conserved in most (>95%) *E. coli* genomes (Fig. 5); with the exceptions of CDS146 (92.8%), CDS66 (in-*ompC*; 89.8%), CDS57 (in-*marR*; 87.1%), and CDS87 (in-*ypjA*, 44.8%), which appear to show some lineage restriction and might suggest these are more recently acquired or evolved genes (Supplementary Fig. S11). We were unable to ascertain any conservation data for the shortest gene, CDS42, and this was excluded from subsequent analysis. For eight of the small genes, we identified putative homologs outside *E. coli* (Supplementary Data S1).

**Figure 6.**
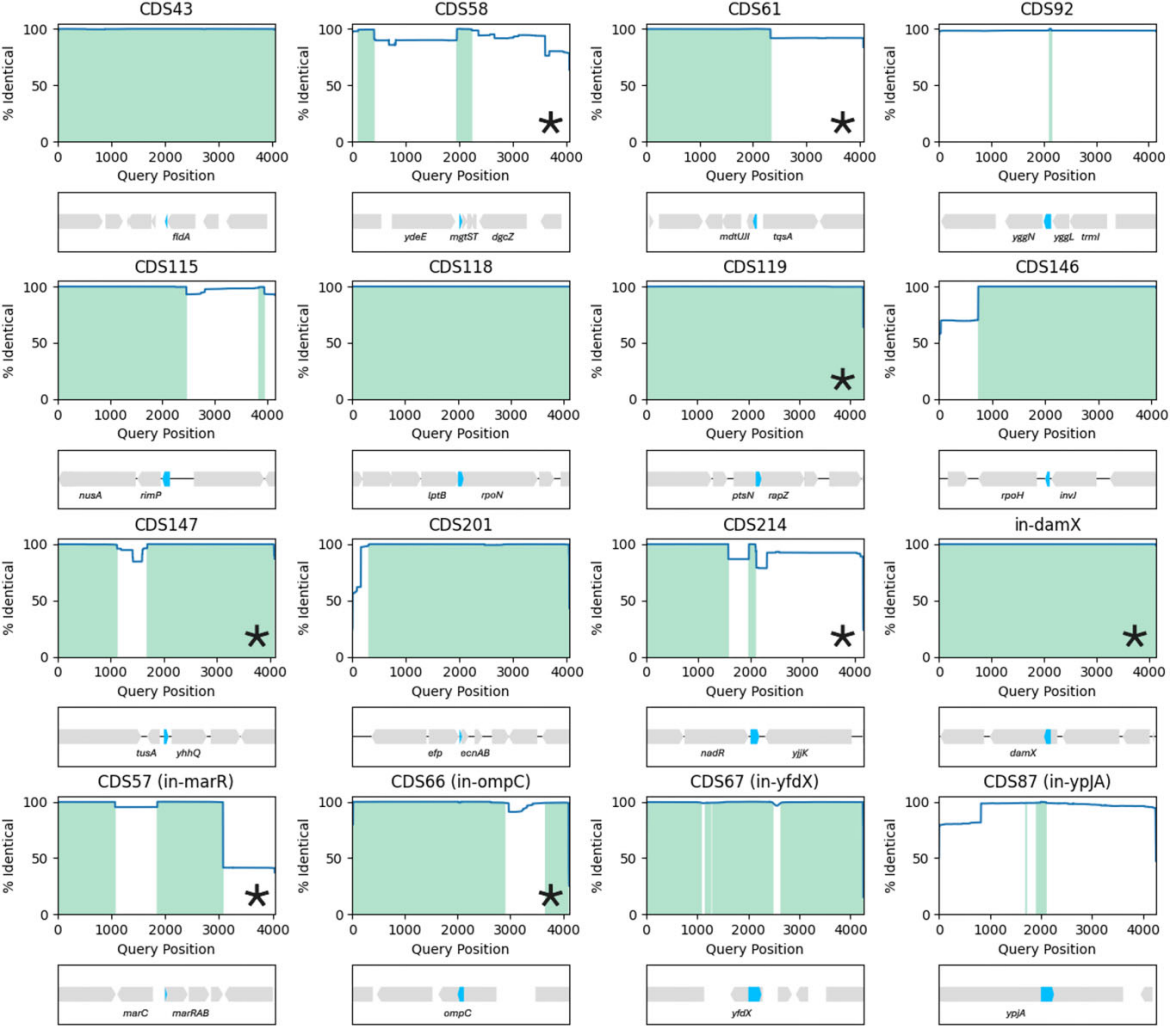
Nucleotide identity plots of gene neighbourhoods. Nucleotide sequence identity 2 kb up- and down-stream of each CDS from the *E. coli* BW25113 reference genome compared with 2 kb surrounding homologs identified within the *E. coli* database of 227 873 genomes. Each plot shows the % of genomes (*y*-axis) that have coverage along the reference genome query sequence (*x*-axis), with the *E. coli* BW25113 reference sequence indicated below. Regions of 100% identity are shaded. Annotated genes are shown in grey, candidate new genes are highlighted in blue, the *mgrR* RNA (downstream *mgtST*) is coloured in green. CDS additionally detected by western blot are indicated with a star.Supplementary Information.

To establish whether the small genes are co-conserved with their gene neighbours, we reviewed whether the genetic synteny of the CDSs was preserved across the different *E. coli* isolates. A 4 kb window (2 kb up- and down-stream) surrounding each CDS was extracted from the *E. coli* BW25113 genome as a reference. The 4-kb region was compared by blastn to the corresponding *E. coli* database strains with identified homologs, to establish whether local gene neighbourhoods were maintained. Most CDS were conserved in ≥ 99% of isolates with at least one gene-neighbour indicating co-conservation (Fig. 6). For some genes, e.g. CDS43 neighbouring *fldA*, or CDS119 neighbouring *ptsN* and *yhbJ*, gene synteny was preserved along the full 4 kb region. Whereas CDS58 was more strongly co-conserved with the neighbouring *mgtST* operon than neighbouring *ydeE*, indicating the CDS58 is part of the *mgtST* operon, and in a subset of *E. coli* isolates (11.7%) this operon may be located elsewhere in the genome. While CDS115 was more strongly co-conserved with *rimP* suggesting a leader sequence for the *rimP-nusA* operon. Genes are often co-localised with functionally related genes and the level of conserved organisation might hint at the potential functions of these newly identified genes. Altogether the new small genes were co-conserved with genes with a range of biological functions, highlighting the wide degree of biological potential that may be overlooked by excluding small proteins.

## Discussion

PIRT-Seq is a genetics-based method for the identification of protein coding sequences on a whole-genome scale, independently of genome annotation. It makes use of saturating mutagenesis that can be achieved with a mini-Tn*5* system coupled with a translation reporter and amplicon sequencing to identify the transposon position, thus revealing translated coding sequences. We applied this method to *E. coli* as there is a wealth of annotation information available for this organism. Altogether 92.52% of insertions were identified within known annotated genes, serving as a useful internal positive control and demonstrating the functionality of PIRT-Seq. The remaining 7.48% of insertions were not consistent with annotated genes and were explored further. We detected translation in 215 ORFs that also contained putative start codons (Supplementary Table S2); the majority of these putative CDS were <100 amino acids, suggesting further as yet uncharacterised small proteins within *E. coli*. The *E. coli* genome has been highly scrutinised for the identification of novel small proteins by various methods [9, 44, 69], yet PIRT-Seq still revealed >200 putative new coding sequences. We validated the endogenous expression of small proteins detected exclusively by this approach using western blot analysis, and confirmed detection of nine out of 17 of these CDSs, three of which are encoded within overlapping genes. We present this as a high-throughput method complementary to Ribosome-trapping or mass spectrometry-based approaches for detecting protein expression.

While the three protein identification methods share many identified proteins in common (Supplementary Fig. S6), there are clearly method-dependent differences. One limitation of this method is that essential genes, or genes that are not easily disrupted, e.g. those bound by DNA-binding proteins, will be less well-represented within the transposon mutant pool, limiting their opportunities for detection. Secondly, shorter genes will also be represented by fewer translation-fusion mutants than longer genes. Our data suggests that both of these limitations may at least in part be overcome using a mini-Tn*5* system that can achieve near saturated mutagenesis. While there are some early reports of a preferred sequence motif for Tn*5* insertion, a more recent study analysing multiple Tn*5* datasets in different backgrounds found this is not a major constraint for Tn*5* transposon insertion when constructing near-saturated libraries [76]. However, we can’t exclude the possibility that there are additional localised constraints that might limit transposon insertion and therefore limit the power of a transposon-mutagenesis based translation screen.

Another explanation for method-dependent differences in protein identification are the experimental conditions. For example, the approach here involves lethal selection on an agar plate and requires mature colony formation. This would limit the detection of proteins expressed later in the growth cycle as expression of the kanamycin resistance cassette is needed for the cell to survive. Although we envisage that this might be overcome in future iterations with a non-lethal reporter selection.

Our data was, however, in agreement with the Ribo-Seq data for the identification of several small genes including *mgtT*, *mdtU* and *ysgD*. We also detected translation in alternate reading frames neighbouring these genes (Fig. 3D) and our gene neighbourhood conservation data revealed conserved clusters of genes organised into transcriptional units of small genes, e.g. CDS58-*mgtST*, CDS61-*mdtUJI*, or CDS177-*ysgD*. One explanation for the detection of additional genes not previously identified by Ribo-Seq might be due to detection sensitivity (discussed below) but was in part due to our wider acceptance of putative start codons: where Weaver *et al.* used a conservative shortlist of AUG, GUG and CUG codons [41], we allowed for seven putative start codons, which enabled the positive identification of CDS177 overlapping *ysgD*. However, these data are likely still an underestimate of the total proteome as earlier work using a GFP reporter has shown as many as 47 of the 64 codons (including one canonical stop codon) are able to initiate translation [67]. Ribo-Ret analyses that allow for a wider selection of start codons observe more varied start codon usage [32], although this in part has been attributed to decreased ribosome selectivity due to increased 30S subunit availability.

While this method identified and validated small proteins not identified in previous screens, we note that more candidates (125) were identified within parent genes compared to intergenic candidates (90). Nested genes are considered rare in prokaryotes. It is possible that some of these have been detected as a result of ribosomal frame shift somewhere between the parent gene start codon and the kanamycin resistance cassette, especially as 116/125 of the nested candidates are translated in the same direction as the parent gene, and the majority of these (98/116) have a + 1 frameshift relative to the parent gene. However, it's worth noting that this is not a widespread phenomenon within the data (92.52% of data are the correct reading frame for annotated genes) and detection of the out-of-frame candidates are supported by multiple independent transposon mutants. Whether these represent programmed ribosomal frame shifting events with biological function remains to be explored.

One recent study combining the analysis of stalled ribosomes at start and stop codons identified almost 400 putative CDSs, however this high number was attributed in part to pervasive translation [44]. While pervasive translation, described as the spurious translation of non-functional coding sequences, could contribute to false positive translation-detection in our screen, we found that the recovered translation-fusion mutants of known genes was quite low relative to the maximum possible translation-fusion mutants (27.5%: the number of detected correct-RF insertions within annotated genes/the total number of correct-RF insertions for those genes) suggesting active translation alone is insufficient for positive translation-fusion detection, and that a degree of mRNA stability is required for successful aminoglycoside phosphotransferase expression.

It is difficult to differentiate translational noise from functional translation, however, the expression of some of the new CDS were validated by western blotting, thus demonstrating the utility of this method for the identification of novel small proteins. For those we were unable to validate by western blot analysis, some limitations for detection of these proteins by western blot analysis were discussed earlier. Notably, the detection limits of western blot immunoblotting are not established. For example, we did detect faint bands for two of the CDS at the expected migration site with excessive blot contrast enhancement, we deemed these insufficient for validation and excluded them from our data. Additionally, there were CDS identified by the transposon-reporter that we could not validate by western blot yet have conserved coding sequences (inclusive of start and stop codons) outside of *E. coli*, e.g. CDS115, CDS118, and CDS201. It is possible that a transposon mutagenesis approach can offer an improved detection resolution as compared to detection by standard western blot, especially for small proteins that are too transiently or weakly expressed to be detected by standard western blot approaches without purification, as both the transposon translation reporter and SPA-tag detection by western blot inherently rely on the same principles of C-terminal tagging of endogenous proteins.

Finally, a strength of this method is its bp level of resolution. First, this can allow for the detection of exquisitely small proteins, or translation events. Although shorter genes are less likely to be hit with a transposon decreasing their chances of discovery, there is no lower-limit size threshold for the functionality of this method, in contrast with proteomics which rarely detects proteins smaller than 40 aa [29]. Secondly, the bp level of resolution enables the identification of the precise reading frame where translation is occurring, unlike Ribo-Seq methods which infer the reading frame based on nearby predicted start codons. However, due to the nature of the screen, it is impossible to know the initiating start codon, which can confound interpretation of within-gene hits.

While others have previously coupled random transposon mutagenesis to identify coding potential, this study is the first to do so at scale and to validate the findings [47, 77]. As a method it is applicable to any organism that is genetically tractable, and the scope of application is much broader than the system presented here. One obvious adaptation of this method is to replace the reporter marker with a non-lethal selection marker, such as a fluorescent protein, which, coupled with fluorescence activated cell sorting, would enable rapid *in vivo* screening for condition-dependent changes in expression. This would expand the capabilities for small protein detection as one often cited limitation is that small proteins might only be expressed transiently, at low levels, or under specific conditions. With many of these tools still in their relative infancy, and focusing on a limited range of growth conditions, it is likely there are still more small proteins to be discovered in the *E. coli* genome. In particular, nested genes have been gaining increasing interest and traction within the field [30, 78, 79]; with data presented here adding another line of evidence in support of their wider existence. We also detected translation within other unexpected genomic regions, including within a handful of repetitive extragenic palindromic (REP) elements. A phenomenon that has also been reported in another study measuring transcriptomics and proteomics in *E. coli* O157:H7 [80].

Many of the newly identified genes were conserved throughout *E. coli* and often co-conserved with their genetic neighbours, with some genes predicted to have homologs in other genera, adding further support for their biological importance. Small proteins are increasingly being recognised for their diverse biological roles, which often includes the modulation and regulation of larger protein complexes [81]. Our identification and detection of small proteins co-conserved with genes involved in electron transfer (*fldA*), magnesium transport (*mgtST* [82]), ribosome maturation (*rimP* [83]), spermidine efflux (*mdtUJI* [84]), potassium transport (*ptsN* [85]), regulatory RNA stability (*rapZ* [86]), queuosine precursor transport (*yhhQ* [87]) NAD^+^ biosynthesis (*nadR*), and tRNA methyltransferase (*trmI*), among others, suggests further functional contributions of small proteins and highlights the functional importance of small proteins that are still being overlooked.

While this paper was under review, a paper examining the conservation and coding potential of conserved intergenic regions in *Enterobacteriaceae* was published [29]. They identified and validated the existence of several new small proteins in *E. coli* K-12 strain MG1655 demonstrating that the coding potential of *E. coli* K-12 is still not fully realised. They reported the lineage-restricted conservation patterns of young genes, which we observed for some of our CDS (Supplementary Fig. S11). Interestingly they noted the potential for translated intergenic small ORFs with non-canonical start codons and lower translation initiation rates. While we cannot quantify translation-initiation rates in this data, we did observe translation in reading frames with non-canonical start codons. They also explored the predicted structures of small proteins and reported α-helices as the most frequent secondary structure in their data, which we observed here (Supplementary Fig. S10). Lastly, they found that intergenic small ORFs co-occurred with neighbouring genes annotated as ‘transporters’ more frequently than expected by chance. Two of the small proteins identified and validated here (CDS119 and CDS147) are predicted to be α-helical and are co-conserved with their neighbouring genes *ptsN* and *yhhQ* involved in regulating potassium transport and transporting queuosine precursors, respectively. It’s tempting to speculate that CDS119 and CDS147 might modulate their neighbouring encoded proteins, but further work is needed to test this.

Finally, this work highlights that a standardised approach to genome annotation is needed [88, 89], and this has been recognised with the implementation of the NCBI Prokaryote Genome Annotation Pipeline applied to all newly submitted sequence data. However, we found that even the most “up to date” annotation of *E. coli* K-12 strain BW25113 (re-annotated 24-APR-2023) did not include many of the small proteins identified and validated in Ribo-Seq experiments from 5 years ago within the same (or closely related) strains [9]. As such, even for one of the best curated strains, *E. coli* K-12, high-throughput functional genetics screens that rely on genome annotations for genotype-phenotype results still overlook small proteins.

## Supplementary Material

gkaf774_Supplemental_Files

## Data Availability

Sequence data are available at the European Nucleotide Archive repository under the project accession: PRJEB89969, in .fastq format with the sample barcodes and transposon sequences removed.
