# Supplementary material for: PIRT-Seq: a high-resolution whole-genome assay to identify protein-coding genes
Source: Nucleic Acids Res. 2025 Aug 13;53(15):gkaf774. doi: 10.1093/nar/gkaf774 (PMC12350097; doi:10.1093/nar/gkaf774)
Supplement: gkaf774_Supplemental_Files [file gkaf774_supplemental_files.zip › S. Table 3 oligos.docx]

***Supplementary Table 3. Oligonucleotides used in this study***

| **Name** | **Sequence** (5′ to 3′) | **Comments** |
| --- | --- | --- |
| Construction of the transposon and cloning | | |
| IR_Kan_Tn.F | ctgtctcttatacacatctcgAGCCATATTCAACGGGAAACG | Stitch PCR |
| Chlor-Tn-HR.R | ggaacctcttacgtgccgatcaacgtctcattttcgccaattacttacTTAGAAAAACTCATCGAGCATC | Introduces stop codons in all 3 RF |
| Cm.start.F | gaaaatgagacgttgatcggcacgtaagaggttcc | Stitch PCR |
| IR.Cm.end.R | ctgtctcttatacacatctaccgggtcgaatttgc |  |
| HindIII-kan-chlor.F | ataccgAAGCTTgctgtctcttatacacatctcgAGCCATATTCAACGGGAAACG | For cloning KC into pUC19 |
| BamHI-kan-chlor.R | ataccgGGATCCctgtctcttatacacatctaccgggtcgaatttgc |  |
| P-ME-kan-chlor_Tn.F | Phos-ctgtctcttatacacatctcgAGCCATATT | Amplification of the Tn to make transposome |
| P-ME-kan-chlor_Tn.R | Phos-ctgtctcttatacacatctaccgggtcga |  |
| lacZa_ATG>AGG.F | ACACAGGAAACAGCTAGGACCAGGATTACGCCA | Mutation of LacZa start codon |
| lacZa_ATG>AGG.R | TGGCGTAATCCTGGTCCTAGCTGTTTCCTGTGT |  |
| lacZa_cloning_check_up.F | GTGAGTTAGCTCACTCATTAGGC | Primer for Sanger sequencing confirmation |
| Transposon sequencing primers | | |
| KC_PCR1_k.F | GAATTTAATCGCGGCCTCGAGCAAGACG | PCR1: amplification enrichment of Tn-gDNA junctions |
| PCR1.R | GACTGGAGTTCAGACGTGTGCTCTTCCGATC | Homology to. NEBNext adapter sequence |
| KC_k_6.1 | AATGATACGGCGACCACCGAGATCTACACTCTTTCCCTACACGACGCTCTTCCGATCTCGTACGCGTTGAATATGGCTCGAGATGTGTA | PCR2: nested PCR, prepares tn-gDNA junctions for sequencing and introduces barcode (underlined) |
| KC_k_7.4 | AATGATACGGCGACCACCGAGATCTACACTCTTTCCCTACACGACGCTCTTCCGATCTTAGCTAGCGTTGAATATGGCTCGAGATGTGTA |  |
| KC_k_8.2 | AATGATACGGCGACCACCGAGATCTACACTCTTTCCCTACACGACGCTCTTCCGATCTGCATGCATCGTTGAATATGGCTCGAGATGTGTA |  |
| KC_k_9.2 | AATGATACGGCGACCACCGAGATCTACACTCTTTCCCTACACGACGCTCTTCCGATCTATCGATCGACGTTGAATATGGCTCGAGATGTGTA |  |
| Construction of SPA tagged mutants | | |
| CBP_stitch.F | AAGAGAAGATGGAAAAAGAATTTCATAG |  |
| CBP-stitch-Kan.R | gtcgacggatccccggaatCTACTTGTCATCGTCATCCTTG |  |
| P1_primer.F | Attccggggatccgtcgac | (Baba et al., 2006) |
| P2_primer.R | tgtaggctggagctgcttcg |  |
| CDS42_DW_tag.F | aggcgattgccgcgaagatgaacatgcgcacgaaggcaaataagccagccAAGAGAAGATGGAAAAAGAATTTCATAG |  |
| CDS42_DW_tag.R | tatttcccttgcataaaaaagccaacccgcaggttggcttttctcgttcatgtaggctggagctgcttcg |  |
| CDS43_DW_tag.F | aattctcaatgcctgatgtgatgcggcgtagactcatgtctacgccgtatAAGAGAAGATGGAAAAAGAATTTCATAG |  |
| CDS43_DW_tag.R | agcaaaagttacaaatttgtagcaattattttgattggcattatctattatgtaggctggagctgcttcg |  |
| CDS58_DW_tag.F | tgaaaattaaggtaagcgaggaaacacaccacaccataaacggaggcaaaAAGAGAAGATGGAAAAAGAATTTCATAG |  |
| CDS58_DW_tag.R | taaaattattcccagtacggccataaaaacattcatattacccagcattatgtaggctggagctgcttcg |  |
| CDS61_DW_tag.F | aactaccgaggacaattatcatccgcgatgacgagaagcaacactgcggaAAGAGAAGATGGAAAAAGAATTTCATAG |  |
| CDS61_DW_tag.R | taaatgtagaaaataacgtcctgaacaaattgtccataatattacaattatgtaggctggagctgcttcg |  |
| CDS92_DW_tag.F | catggaaaacgcgtactttgttatcaatctggggccagcaaatgctggccAAGAGAAGATGGAAAAAGAATTTCATAG |  |
| CDS92_DW_tag.R | tgccgccagcagcattttgcgcatcatagtcttccctcaagaaaaaatcatgtaggctggagctgcttcg |  |
| CDS115_DW_tag.F | ccccgatttatcggggttttttgttatctgactacagaataactgggcttAAGAGAAGATGGAAAAAGAATTTCATAG |  |
| CDS115_DW_tag.R | tttgctctaatgtggacaagcccacccccaagacataaaaaaagggcctatgtaggctggagctgcttcg |  |
| CDS118_DW_tag.F | tagcaggagagtacgattctgaacatgaagcaaggtttgcaactcaggctAAGAGAAGATGGAAAAAGAATTTCATAG |  |
| CDS118_DW_tag.R | acagacgaattgcctgttggagctgtggcgtcatcgccagttgttggctatgtaggctggagctgcttcg |  |
| CDS119_DW_tag.F | attcggtaatgtctcttttagacgttgtgaggagaaacagtacatggtacAAGAGAAGATGGAAAAAGAATTTCATAG |  |
| CDS119_DW_tag.R | acgcagggcgacagatttacctgaacctgaacgtccgctgacgatcatcatgtaggctggagctgcttcg |  |
| CDS146_DW_tag.F | gtaataatcaatttcccctccggcaaaacgccaatccccacgcagattgtAAGAGAAGATGGAAAAAGAATTTCATAG |  |
| CDS146_DW_tag.R | aacatacccgatttttatgatattggaatagctattttgacagtttattatgtaggctggagctgcttcg |  |
| CDS147_DW_tag.F | agtatcatgcggcggctcgaaaaaagggtaagcacgttattatgttaaggAAGAGAAGATGGAAAAAGAATTTCATAG |  |
| CDS147_DW_tag.R | ttgattaaccattggggtgagggaacccaatacgtacgacacgtctgttatgtaggctggagctgcttcg |  |
| CDS201_DW_tag.F | cggttgtggtgcggcctgcaggctgcaccatcacttattcaggtcagagaAAGAGAAGATGGAAAAAGAATTTCATAG |  |
| CDS201_DW_tag.R | cgtgagcagcgtgctggcaagcaaaacaagaacgataaggcgtttcatcatgtaggctggagctgcttcg |  |
| CDS214_DW_tag.F | tagaccggaaacggtgttcacgccgcatccggcattcggtgctcaatgccAAGAGAAGATGGAAAAAGAATTTCATAG |  |
| CDS214_DW_tag.R | aatacgttgtaaaactgtaggcctgataagacgcggtaagcgtcgcatcatgtaggctggagctgcttcg |  |
| IN-ompC_DW.F | cgctgcttacatcggtaacggcgaccgtgctgaaacctacactggtggtcAAGAGAAGATGGAAAAAGAATTTCATAG |  |
| IN-ompC_DW.R | ggtctgggtgtactgagcagccaggtagatgttgttagcgtcgtatttcatgtaggctggagctgcttcg |  |
| IN-marR_DW.F | tgtgaaaagtaccagcgatctgttcaatgaaattattccattgggtcgctAAGAGAAGATGGAAAAAGAATTTCATAG |  |
| IN-marR_DW.R | cagatactcgttaagcaggcgatctttcttctgattaaccatatggattatgtaggctggagctgcttcg |  |
| IN-damX_DW.F | cgaagaagaaattgacgaatccgaagatgaaaccgtggatgaagagcgcgAAGAGAAGATGGAAAAAGAATTTCATAG |  |
| IN-damX_DW.R | agaagcgggtttactggctgcttttttgcgcttacgcggacgacgctctatgtaggctggagctgcttcg |  |
| AS-yfdX_DW.F | taccggagcattatcagcagcccatacagttgaagatgccagaattgctgAAGAGAAGATGGAAAAAGAATTTCATAG |  |
| AS-yfdX_DW.R | gttaaagaatgaggtaagtatgaaacgtttaattatggccacgatggtcatgtaggctggagctgcttcg |  |
| AS-ypjA_DW.F | gagagcttgttgtatccttcgatatcagtccctgcacccgtgttggcaacAAGAGAAGATGGAAAAAGAATTTCATAG |  |
| AS-ypjA_DW.R | ataccggcggtattgcacatggggttaaccaggagacgggcagtgctttatgtaggctggagctgcttcg |  |
| PCR Check of SPA-tagged mutants | | |
| CDS42_check.F | aatttagtgatgattccatcg |  |
| CDS42_check.R | ttgtgataaagtctggcagga |  |
| CDS43_check.F | atgacgaccactttgtcggtc |  |
| CDS43_check.R | Cggtattgttatcagtcatgc |  |
| CDS58_check.F | gatcattgctgcgtgggtgct |  |
| CDS58_check.R | tcaacgattcgttatcagtgc |  |
| CDS61_check.F | ccattgagcgtgatgatcgg |  |
| CDS61_check.R | ccatttcattgacagcgtacc |  |
| CDS92_check.F | gtacatcggaagaacagattg |  |
| CDS92_check.R | gtgacgctgcactggtagtc |  |
| CDS115_check.F | ctcggtacaccaaatcccagc |  |
| CDS115_check.R | aactggcgcagtaatcatctc |  |
| CDS118_check.F | caacgtgcgtgaaacactggc |  |
| CDS118_check.R | Tgtaaatggtgtcccaactgg |  |
| CDS119_check.F | cattccgcatggcaaactgga |  |
| CDS119_check.R | agatcgggtaacaacactacg |  |
| CDS146_check.F | ccagcgcgatacatcctgcc |  |
| CDS146_check.R | caacaagtccacgttgcagga |  |
| CDS147_check.F | gatcgtcggcgataatcagc |  |
| CDS147_check.R | gctgaaccagatagttactgg |  |
| CDS201_check.F | ccctgtctactggcgctgtgg |  |
| CDS201_check.R | aatgtcttcaccaacgccacg |  |
| CDS214_check.F | acttgctggtggagatgctcg |  |
| CDS214_check.R | ttggttcctcgaccgtatcgc |  |
| IN-ompC_check.F | actacggtcgtaactacgg |  |
| IN-ompC_check.R | gatatcttcgtcgtcgtagc |  |
| IN-marR_check.F | tttagctagccttgcatcg |  |
| IN-marR_check.R | aggtcgaccgacaatacc |  |
| IN-damX_check.F | cgatcgtcgtactggtcg |  |
| IN-damX_check.R | ctgatcggtcgcattgc |  |
| AS-yfdX_check.F | tggcttagcgaactttgc |  |
| AS-yfdX_check.R | aatgaccgttgctctctg |  |
| AS-ypjA_check.F | cttaccgccagtatcaatgg |  |
| AS-ypjA_check.R | aattatggcatagccacagg |  |

**References**

Baba, T., Ara, T., Hasegawa, M., Takai, Y., Okumura, Y., Baba, M., Datsenko, K. a, Tomita, M., Wanner, B.L., Mori, H., 2006. Construction of *Escherichia coli* K-12 in-frame, single-gene knockout mutants: the Keio collection. Mol. Syst. Biol. 2, 2006.0008. https://doi.org/10.1038/msb4100050
