# Supplementary material for: PIRT-Seq: a high-resolution whole-genome assay to identify protein-coding genes
Source: Nucleic Acids Res. 2025 Aug 13;53(15):gkaf774. doi: 10.1093/nar/gkaf774 (PMC12350097; doi:10.1093/nar/gkaf774)
Supplement: gkaf774_Supplemental_Files [file gkaf774_supplemental_files.zip › S.Data.1_Protein_homolog_alignments.docx]

**Amino acid alignment of conserved sORFs**

**Extended Data Legend:** Potential homologues identified in other species of eubacteria or plasmids are shown. Species genera are shown in each alignment, with the species names being listed below the alignment. Amino acids in red are potential start codons. Stop codons are identified with a ‘#’. Amino acid agreement between species is denoted by a ‘*’ to indicate complete agreement between sequences at the position, ‘:’ to indicate strong biochemical similarity between residues at the position and ‘.’ to indicate some biochemical similarity between residues at the position. Sequences were identified using the microbial database tBLASTn search (blast.ncbi.nlm.nih.gov/Blast.cgi?PROGRAM=tblastn&PAGE_TYPE=BlastSearch&BLAST_SPEC=MicrobialGenomes&LINK_LOC=blasttab&LAST_PAGE=blastn) and alignments were generated using ClustalW (www.genome.jp/tools-bin/clustalw). Further description of search criteria is provided in the Materials and Methods.

**CDS61**

**Escherichia^1^ MIQIRRVQLPRTIIIRDDEKQHCG#**

**Salmonella^2^ MIQIRRVQLPRTIIIRDDEKQHCG#**

**Citrobacter^3^ MIQIRRVQLPRTIIIRDDEKQHCG#**

**Enterobacter^4^ MVQIWRVQLPRTILIRKDEKQHRG#**

***:** ********:**.***** ***

^1^*Escherichia coli* str. K-12

^2^*Salmonella enterica* str. SA20021456

^3^*Citrobacter freundii complex sp. CFNIH2*

^4^*Enterobacter sichuanensis* str. SGAir0282

**CDS115**

**Escherichia^1^ VKTSAFVVGFEKILLESAPDRSCGYRVQLYKAPIYRGFLLSDYRITGL#-----------**

**Citrobacter^2^ ---------LKKILSEGSPDRSCGYRVQLSKAPIYRGFLLSDYRITGL#-----------**

**Salmonella^3^ ---MSFVAGFEKILSEGTPDRRCGYRVQLSKAPIYRGFLLSDYRITGL#-----------**

**Klebsiella^4^ VRTRSFVAGFERILLEGVPDRNCGLRVQLSKAPIYRGFLLSDFRITGLYALFLCLGGGFV**

**:::** *. *** ** **** ************:*******

**Escherichia^1^ --------------**

**Citrobacter^2^ --------------**

**Salmonella^3^ --------------**

**Klebsiella^4^ HIRAKINRDAHCAS#**

^1^*Escherichia coli* str. K-12

^2^*Citrobacter amalonaticus* str. FDAARGOS_1489

^3^*Salmonella enterica subsp. enterica serovar Typhi* str. 11

^4^*Klebsiella aerogenes* str. G7

**CDS118**

**Escherichia^1^ VYTLGKTSDSDRVEVCDVLAGEYDSEHEARFATQA#**

**Salmonella^2^ VYILGKTSDSDRVEGYAALAGENDSEYEARFATQA#**

**Klebsiella^3^ ---LGKTSDSDRVEVTDVLAGDIYPEHEARFAIKA#**

************* .***: .*:***** :***

^1^*Escherichia coli* str. K-12

^2^*Salmonella enterica subsp. enterica serovar Typhimurium* str. LT2

^3^*Klebsiella quasivariicola* str. 08A119

**CDS119**

**Escherichia^1^ MPSTTNRWTYFLPCWCRQTKLKRTCILCRWWRNVWRTKPSAAVYAQPRAMKSCIKSLRIP**

**Citrobacter^2^ MPLIINRSICFSPCWCPQIRPKRICIHCRWSLNVWRIKPSAAVCAPRRVTKSCIKSSLTP**

**Enterobacter^3^ MPLITSPSIFSSRCWCLPTRRKPICIRCRWLPNVWPIKPFAADCARPKVMKSSIRLSLKQ**

**Klebsiella^4^ MLSTINPSICCLPFLSRPIKLKRICIRSRWWRNVWQIKLFAAACALRKVMKSCMKSLLKR**

**Raoultella^5^ MPSTTSRSICCLRFWSRLTRPKPICIRCRWSQNDWRIKRFAAACARRKAMKSCMRSSPKQ**

**Kluyvera^6^ MLSTISLLIYCLRFWCPQTRPKPTCIRFPWWRNVWRTKLFAVVCVLRRVTKSCIKSSPIQ**

**Erwinia^7^ MPLIISLWICCLPCWCRQISVKRTCTHFHWSPNAWQIKPFCVVYALHRVMKNCIKSSRKI**

**Pantoea^8^ MPSITSRSICCLPYWCRLTSAKRTCTPFHWWQNVWQIKRSAAVCARRRVMKSSTPLLRKS**

*** . . * * * * * * .. . :. *..**

**Escherichia^1^ KVLRMKRSYSVMSLLDVVRRNSTWY#**

**Citrobacter^2^ KVERMRHNHPMAS---VVRRNGTWY#**

**Enterobacter^3^ KAIRMMHNQEMAFTSVVLRRNGTWC#**

**Klebsiella^4^ EVIMRRNGSRTLLFF-VLRRNGTWY#**

**Raoultella^5^ EATMTYNGRRPLPFI-VLRRNGTWY#**

**Kluyvera^6^ KLTMMRNG-----HHIVLRRNGTWY#**

**Erwinia^7^ TIGSNRAVIYPQSLA#----------**

**Pantoea^8^ RQSSNASLPAGCRF#-----------**

^1^*Escherichia coli* str. K-12

^2^*Citrobacter koseri ATCC BAA-895*

^3^*Enterobacter sichuanensis* str. SGAir0282

^4^*Klebsiella michiganensis* str. THO-011

^5^*Raoultella planticola* str. FDAARGOS_64

^6^*Kluyvera intermedia* str. N2-1

^7^*Erwinia pyrifoliae* str. EpK1/15

^8^*Pantoea deleyi* str. LMG24200

**CDS147**

**Escherichia VKINHCIVNVKQYHAAARKKGKHVIMLR----**

**Enterobacter VKISHCKVSSKQYHAAALLGSLTPNINQKGTI**

*****.** *. ******* . : :**

^1^*Escherichia coli* str. K-12

^2^*Enterobacter hormaechei* str. FDAARGOS 1433

**CDS201**

**Escherichia^1^ -----VVRPAGCTITYSGQR#**

**pKlebsiella^2^ -----VVRPAGCTITYSGQR#**

**Enterobacter^3^ -----VVRPAGCTITFSGQR#**

**Streptomyces^4^ LISTTVHRPSGCTTTASGSR#**

**Kitasatospora^5^ LISTTVQRPP-CTTTASGSR#**

**Nocardia^6^ LISTTWNWPSICSATTSGSR#**

***. *: * **.***

^1^*Escherichia coli* str. K-12

^2^*Klebsiella oxytoca* strain FDAARGOS_1334 plasmid unnamed16

^3^*Enterobacter hormaechei strain RHBSTW-00218*

^4^*Streptomyces californicus strain FDAARGOS_1211*

^5^*Kitasatospora purpeofusca strain NBC_00222*

^6^*Nocardia cyriacigeorgica GUH-2*

**CDS214**

**Escherichia^1^ MSFFDELKTSLEEAVEIKQGLKKPARVTRHEIEDAKAVVDRKRCSRRIRHSVLNA#----**

**Hafnia^2^ MSFFDDLKTSLEEAVDIKNGLKNPARVTRYAIADVKAIREQLNVSQSEMAKALGTSVDTI**

**Lectercia^3^ MNFFDELKTSLEEAVDIKNGTKAPARVTRYEIADVKAIREQLNVSQAEMAKALGTSVDTI**

**Kosakonia^4^ MSFFDELKASLEEAVEIKKGAKKPARVTRYEIADVKAIREQLNVSQGEMAKALGTSVDTI**

**pHafnia^5^ MSFFDELKASLEEAVEIKKGVKAPARVTRYELADVKALRAQLNVTQDEMAKVLGTSLDTI**

**Erwinia^6^ MSFFDELKASLEEAVEIKKGVKAPARVTRYELADVKALRAQLNVTQDEMAKVLGTSLDTI**

***.***:**:******:**:* * ******: : *.**: : . :: ..*.:**

**Escherichia^1^ ----------------------------------**

**Hafnia^2^ KSWETKRRNPTGLAAKVLATIQANPAFFRELASH#**

**Lectercia^3^ KSWESRRRNPTGLAAKVLATIQANPAFFYELAAH#**

**Kosakonia^4^ KSWESGRRNPTGLAAKVLAAIKANPAFFKELSAH#**

**pHafnia^5^ KSWETGRRNPTGLAAKVLATIQANPKFFQELASH#**

**Erwinia^6^ KSWETGRRNPTGLAAKVLATIQANPKFFQELASH#**

^1^*Escherichia coli* str. K-12

^2^*Hafnia alvei* str. PCM_1220

^3^*Leclercia adecarboxylata* str. R25

^4^*Kosakonia pseudosacchari* str. BDA62-3

^5^*Hafnia paralvei* str. AVS0177 plasmid pAVS0177-b

^6^*Erwinia tasmaniensis Et1/99*

**CDS66**

**Escherichia^1^ -MITKVSVSVVRSPAPNVLMLR-TPLLT-----SVTATVLKPTLVV#--**

**Citrobacter^2^ ---------VRQSPAPNVPMSRTTQHVT-----LVTATALKPTPAV#--**

**Atlantibacter^3^ ---VLASVSVVRCPLPNVLLTRTTWLAS-----TVTATTQKPTLAA#--**

**Salmonella^4^ MKSAKASLSAARSPPPNVLPIRTTLLTL-----ACMVTAIAPRSTLAA#**

**Scandinavium^5^ MIWAKASVWVLQCPAPNVLPIRTMHLLVQMALFTVKAIALKPTLAA#--**

**Enterobacter^6^ ---VKASASVVQCLLPNVLTIRTTPQHW------VTAITLKSTLVV#--**

**Klebsiella^7^ MISGKASAQVSRTPTPNVMAIRTASWLK------VVAIMLKPIPVA#--**

**. : *** * . . .**

^1^*Escherichia coli* str. K-12

^2^*Citrobacter rodentium NBRC 105723 = DSM 16636*

^3^*Atlantibacter subterranea* str. LH84-a chromosome

^4^*Salmonella bongori NCTC 12419*

^5^*Scandinavium goeteborgense* str. CCUG 66741

^6^*Enterobacter soli*

^7^*Klebsiella sp. RHBSTW-00464*
