# Supplementary material for: PIRT-Seq: a high-resolution whole-genome assay to identify protein-coding genes
Source: Nucleic Acids Res. 2025 Aug 13;53(15):gkaf774. doi: 10.1093/nar/gkaf774 (PMC12350097; doi:10.1093/nar/gkaf774)
Supplement: gkaf774_Supplemental_Files [file gkaf774_supplemental_files.zip › Supplementary Figures2.pdf]

Supplementary Figures

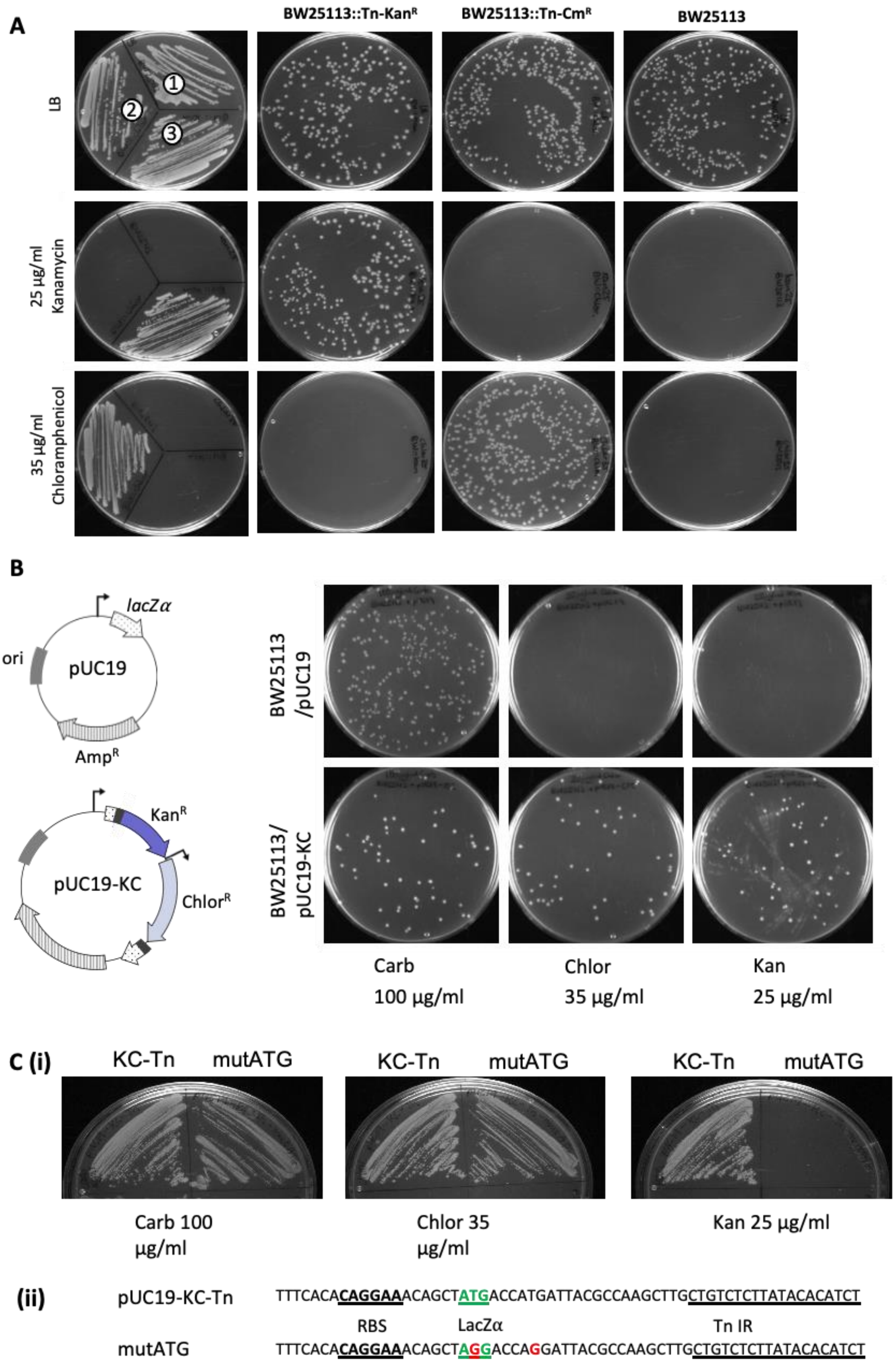

Supplementary Figure 1. Transposon design and functionality

(A) Antibiotic cassette cross-resistance assay. Growth of *E. coli* BW25113 or *E. coli* BW25113 mutagenized at random with a mini-Tn*5* transposon carrying either a kanamycin (Kan^R) or chloramphenicol (Cm^R) resistance cassette on LB agar plates supplemented with antibiotic. The left panel shows the same selection plates with all strains inoculated: (1) *E. coli* BW25113, (2) *E. coli* BW25113::Tn-Cm^R, (3) *E. coli* BW25113::Tn-Kan^R. These transposons formed the basis of the dual selection transposon, stitched together by PCR (excluding the promoter, RBS and start codon of the Kan^R cassette). (B) Translational fusion and expression of the kanamycin resistance gene downstream from and in frame with the LacZα start codon (pUC19-KC). *E. coli* BW25113 was transformed with either the pUC19 plasmid or pUC19-KC on LB supplemented with carbenicillin, chloramphenicol or kanamycin, confirming the LacZα start codon-Tn inverted repeat-Kan^R gene fusion is functional. (C) Single nucleotide polymorphisms were introduced by site directed mutagenesis into the start codon(s) of LacZα, replacing ATG with AGG, to make the pUC19-KC-Tn-mutATG vector. *E. coli* BW25113 with either pUC19-KC-Tn or pUC19-KC-Tn-mutATG were inoculated onto LB agar plates supplemented with carbenicillin, chloramphenicol or kanamycin, confirming a start codon is needed for expression of the Tn-Kan^R gene.

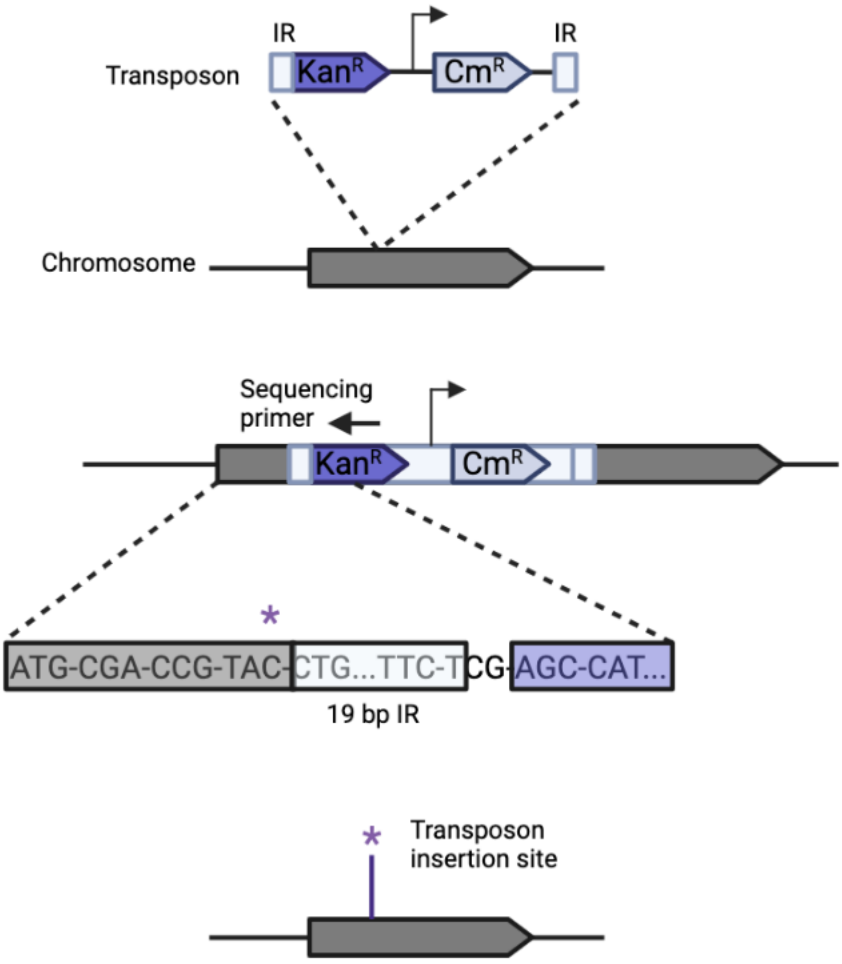

Supplementary Figure 2. Schematic for identification of the transposon insertion site
The transposon inserts into the genome at random. For identification of the transposon insertion site, the transposon-genomic DNA junction is sequenced by sequencing out from the kanamycin resistance gene into the neighboring genomic DNA. The nucleotide immediately adjacent to the 19 bp inverted repeat of the transposon (indicated by an asterisk) is identified as the transposon insertion site. Abbreviations: IR, inverted repeat; Kan^R, kanamycin resistance; Cm^R, chloramphenicol resistance.

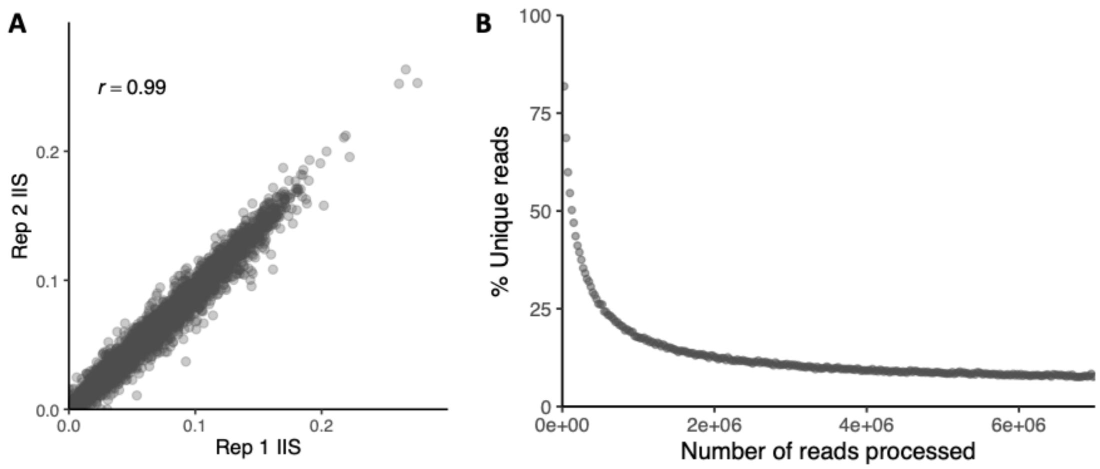

C

Reading Frame	Insertions	Percentage
RF1	61,892	16.72%
RF2	62,034	16.76%
RF3	61,783	16.69%
RF4	61,651	16.66%
RF5	61,219	16.54%
RF6	61,535	16.63%

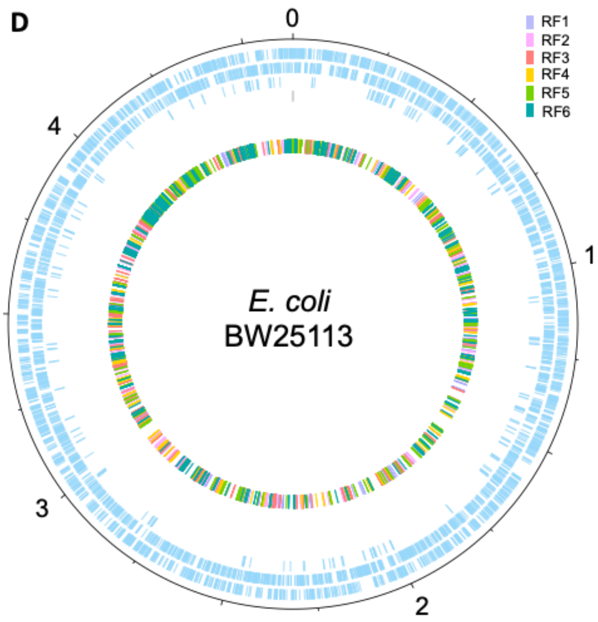

Supplementary Figure 3. Analysis of the reporter transposon library

(A) Comparison of insertion index scores (IIS; the number of insertions per gene normalised by gene length) between sequenced replicates of the input transposon library. (B) Rarefaction plot of the input transposon library. Sub-sampling of transposon-trimmed fastq data, using BBTools, shows the percentage of unique reads with increasing sampling of sequencing data. (C) Proportion of insertions per reading frame (RF) throughout the genome. (D) Chromosomal position of each identified insertion site around the *E. coli* BW25113 chromosome, and therefore translation reporter position, coloured by reading frame (RF). The two outermost tracks in light blue correspond with sense and antisense CDS respectively, with pseudogenes highlighted in light blue inside.

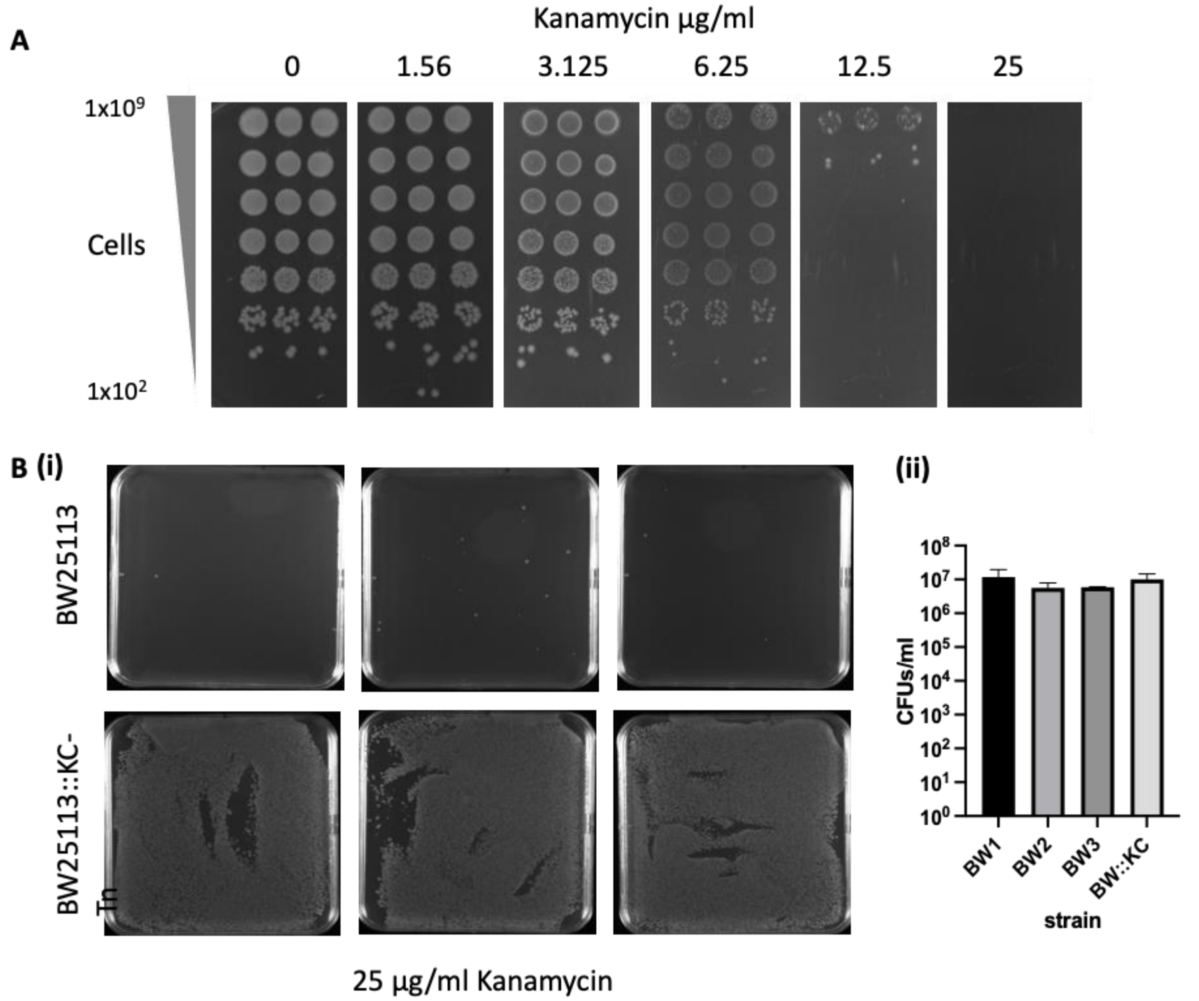

Supplementary Figure 4. Kanamycin selection screening

(A) Identification of a suitable concentration of kanamycin for agar plate selection. *E. coli* BW25113 was grown in triplicate overnight at 37˚C. Samples were normalized to an OD_{600} = 1.00, equivalent to $1x10^9$ cells. Cells were 10-fold serially diluted in LB broth and 5 µl of each dilution was inoculated onto LB agar plates with and without kanamycin at 2-fold decreasing concentrations. Plates were incubated overnight at 37˚C and imaged the following day. (B)(i) Cell cultures of *E. coli* BW25113 (in triplicate) and *E. coli* BW25113::kan^R-Cm^R-Tn (KC-Tn) were normalized to an OD_{600} = 0.01 in LB; 500 µl of this cell culture was inoculated onto square LB agar plates supplemented with 25 µg/ml kanamycin and grown overnight at 37˚C. (ii) In addition, 10-fold serial dilutions of these cultures were inoculated on LB only plates to enumerate the input colony forming units (CFUs) for each sample.

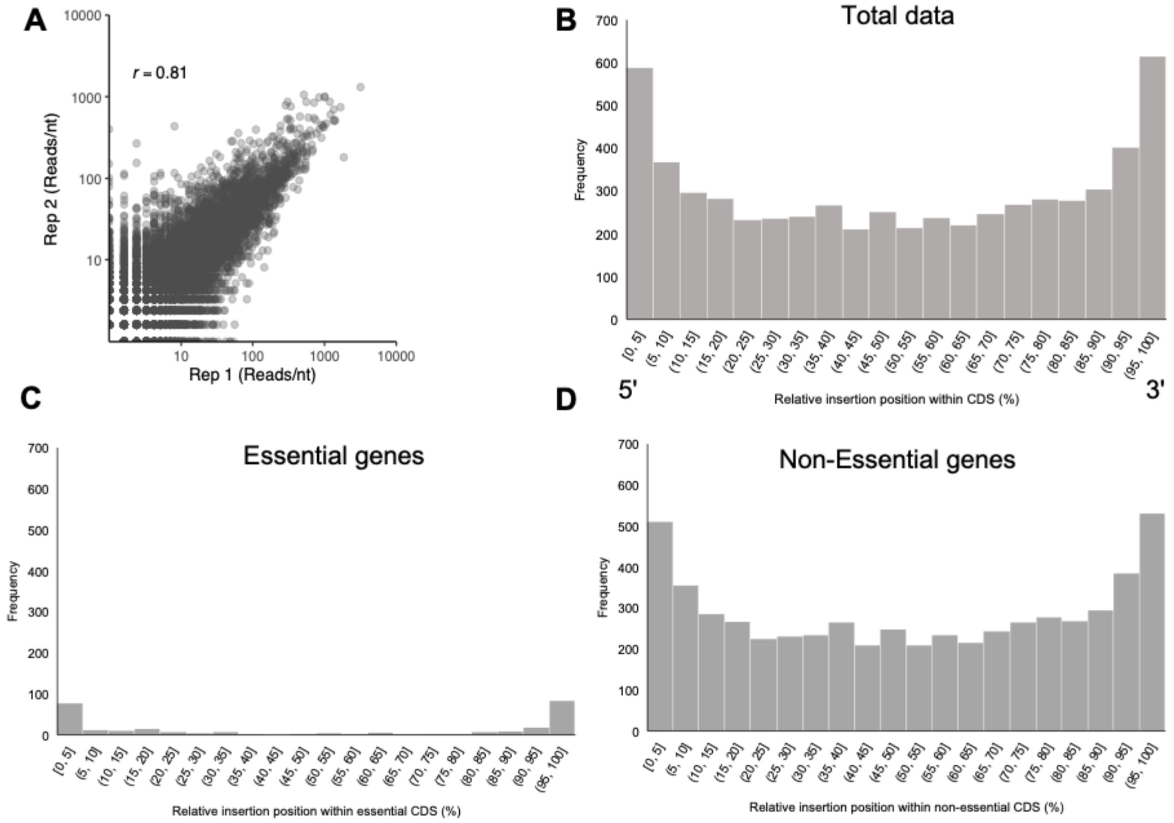

Supplementary Figure 5. **Analysis of transposon mutants following kanamycin selection.** (A) Comparison of the number of reads per nucleotide between replicates of the BW25113::KC-Tn library plated on LB supplemented with kanamycin. (B-D) The position of each insertion within a protein coding sequence relative to the gene start and end taken as a percentage, following kanamycin selection for the total data (B), essential genes (C) and non-essential genes (D), using gene-essentiality data derived from a transposon-insertion dataset in *E. coli* BW25113.

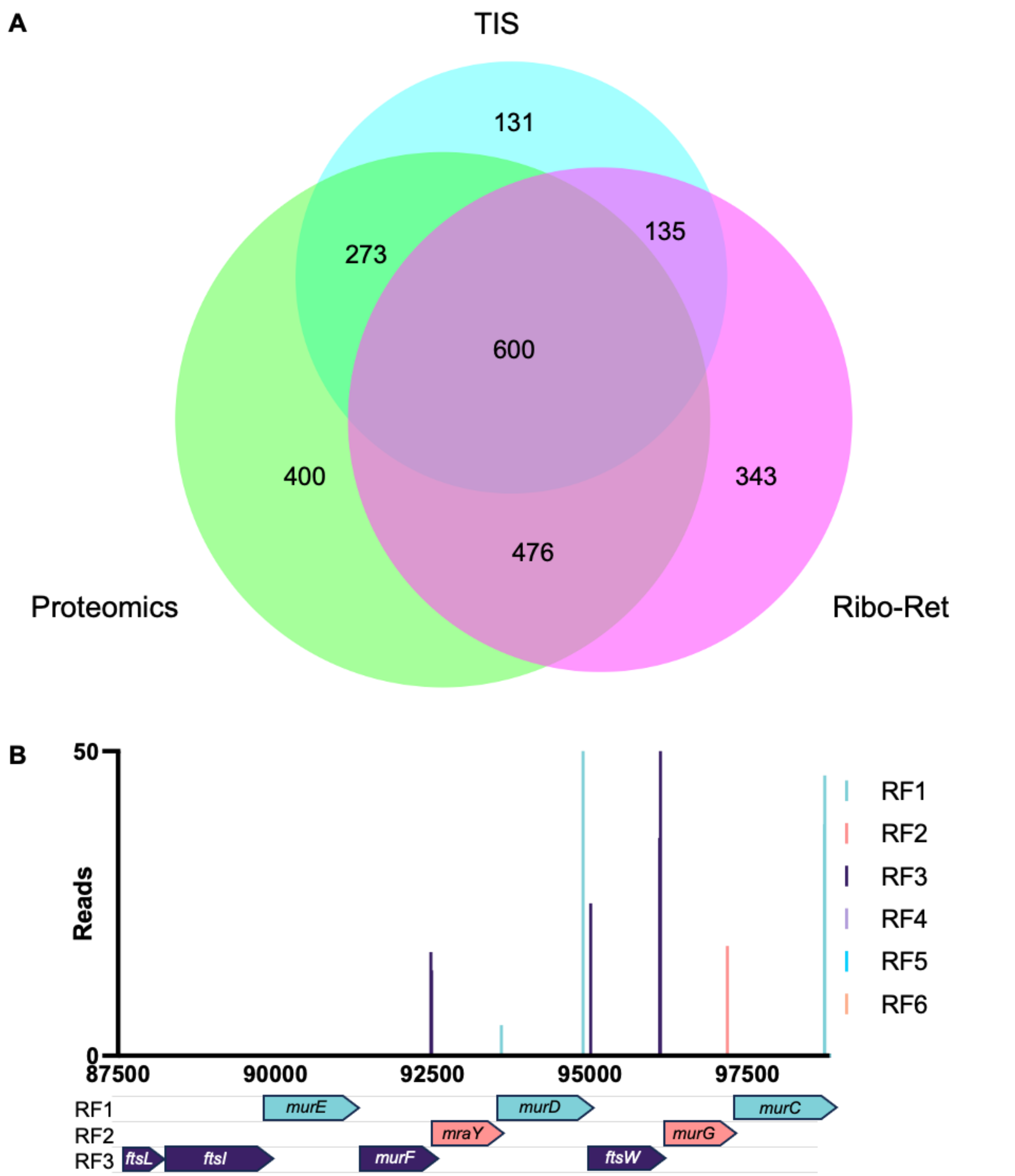

Supplementary Figure 6. Dataset comparison

(A) Comparison of the annotated genes positively identified by different high-throughput methods for the identification of proteins/protein-coding genes on a whole cell scale. All datasets are reported for the *E. coli* K-12 stain BW25113 grown in LB, with the caveat that the transposon-insertion sequencing (TIS) data was exposed to kanamycin, while the Ribo-Ret dataset was a BW25113 derivative with the genotype BW25113Δ*tolC* and grown in LB supplemented with 0.2% glucose prior to exposure to 12.5 µg/ml Retapamulin. TIS method with a Ribo-Ret dataset (Meydan *et al.* 2019) and a Proteomics dataset (Schmidt *et al.* 2016). (B) Expression of essential genes detected by the translation-reporter transposon. Viable translation-fusion events within 5/9 (*murF, murD, ftsW, murG* and *murC*) essential genes of the *mur* operon following selection of the transposon library on kanamycin. Insertions are at

the extreme 5' end of each CDS, coloured according the reading-frame (RF). The presence of 5' transposon insertion events suggests translational read-out from the transposon to maintain expression of downstream essential genes.

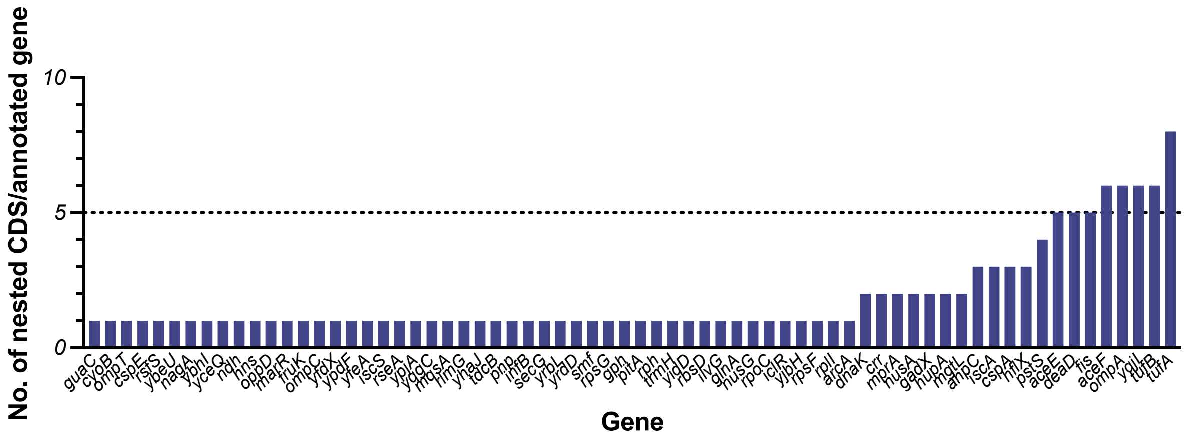

Supplementary Figure 7. Frequency of out-of-frame nested genes within annotated genes
The x-axis lists the annotated genes that an out-of-frame insertion event was identified within, indicative of a nested gene. The height of the bar corresponds with the number of putative nested genes identified by PIRT-seq within these genes.

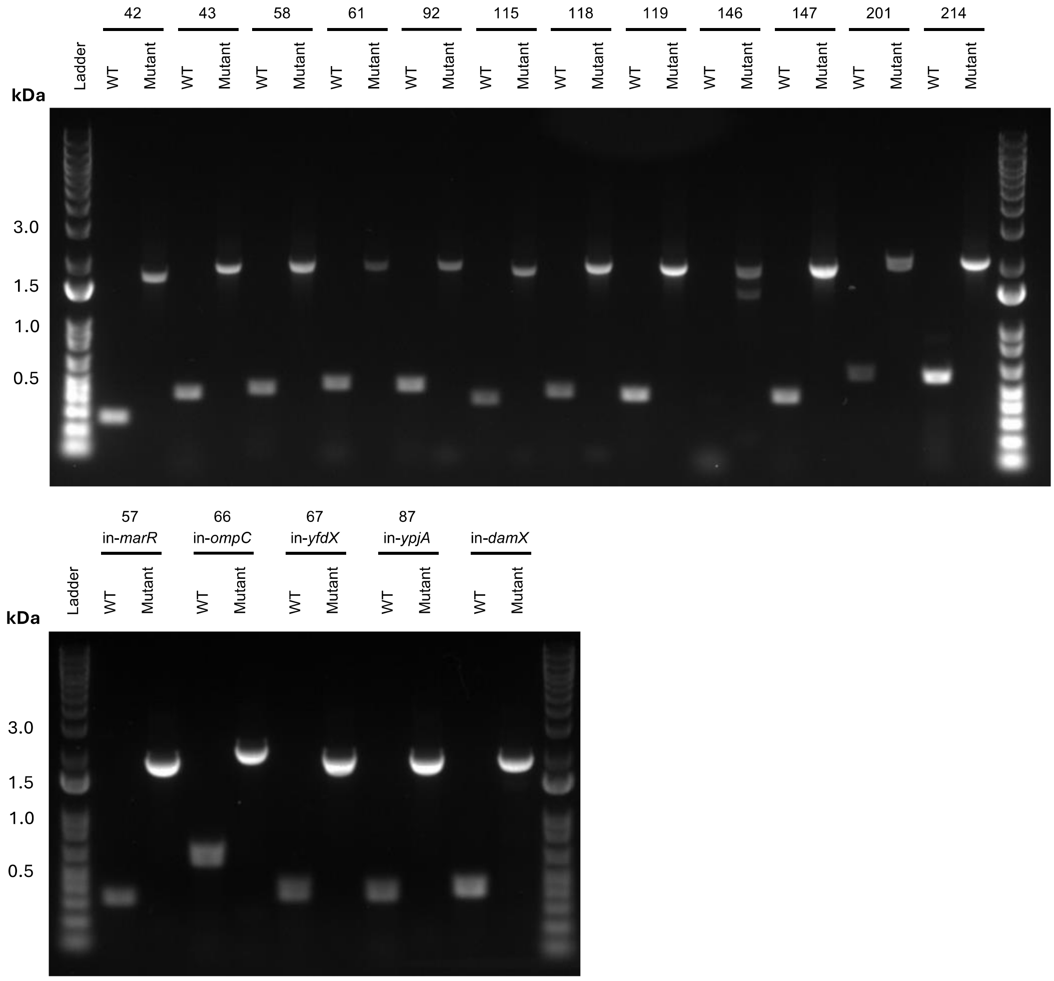

Supplementary Figure 8. PCR validation of SPA-tag introduction

PCR amplification using primers up- and downstream of the stop codon locus of each putative CDS for the parent strain *E. coli* BW25113 (annotated here as WT) and each SPA-tagged construct (annotated here as Mutant). Primer pairs are listed in S. Table 3.

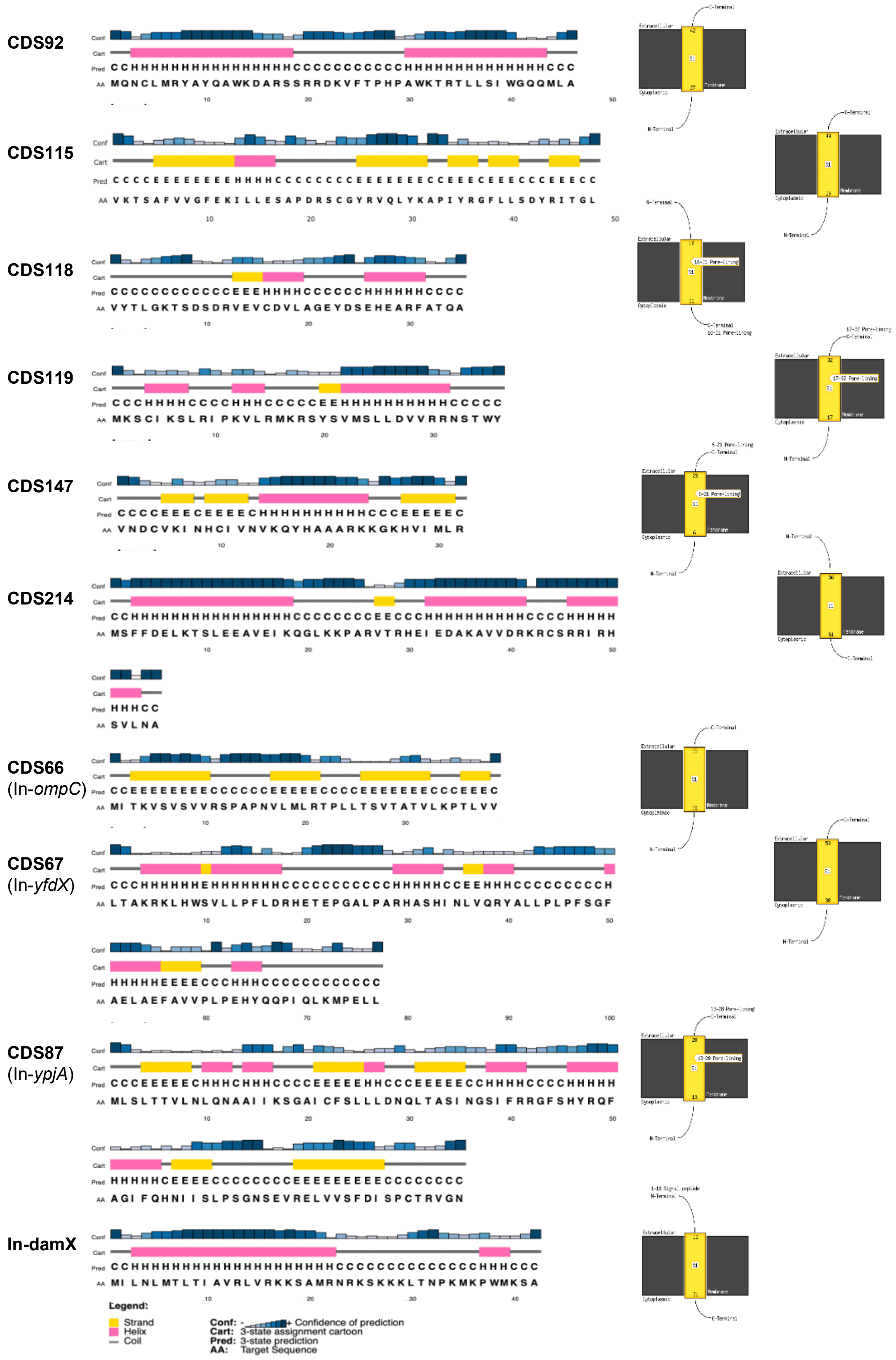

Supplementary Figure 9. Predicted protein structure using PSIPRED
The left column contains the PSIPRED protein structure predictions (Buchan and Jones, 2019), while the right column displays the associated membrane helix prediction using MEMSAT-SVM, where applicable. CDS118 (residues 16-31), CDS119 (17-32), CDS147 (6-21) and

CDS87 (13-28) are predicted to be pore-lining helices with the predicted pore-lining residues listed in parentheses. Each transmembrane helix is oriented with the cytoplasm beneath the figure; CDS118, CDS214 and 'in-damX' are predictedd to have a periplasmic N-terminus, while all others are predicted to have a cytoplasmic N-terminus. CDS42, CDS43, CDS58, CDS61, CDS146, CDS201 and CDS57 were all too short for analysis (minimum size is 30 residues).

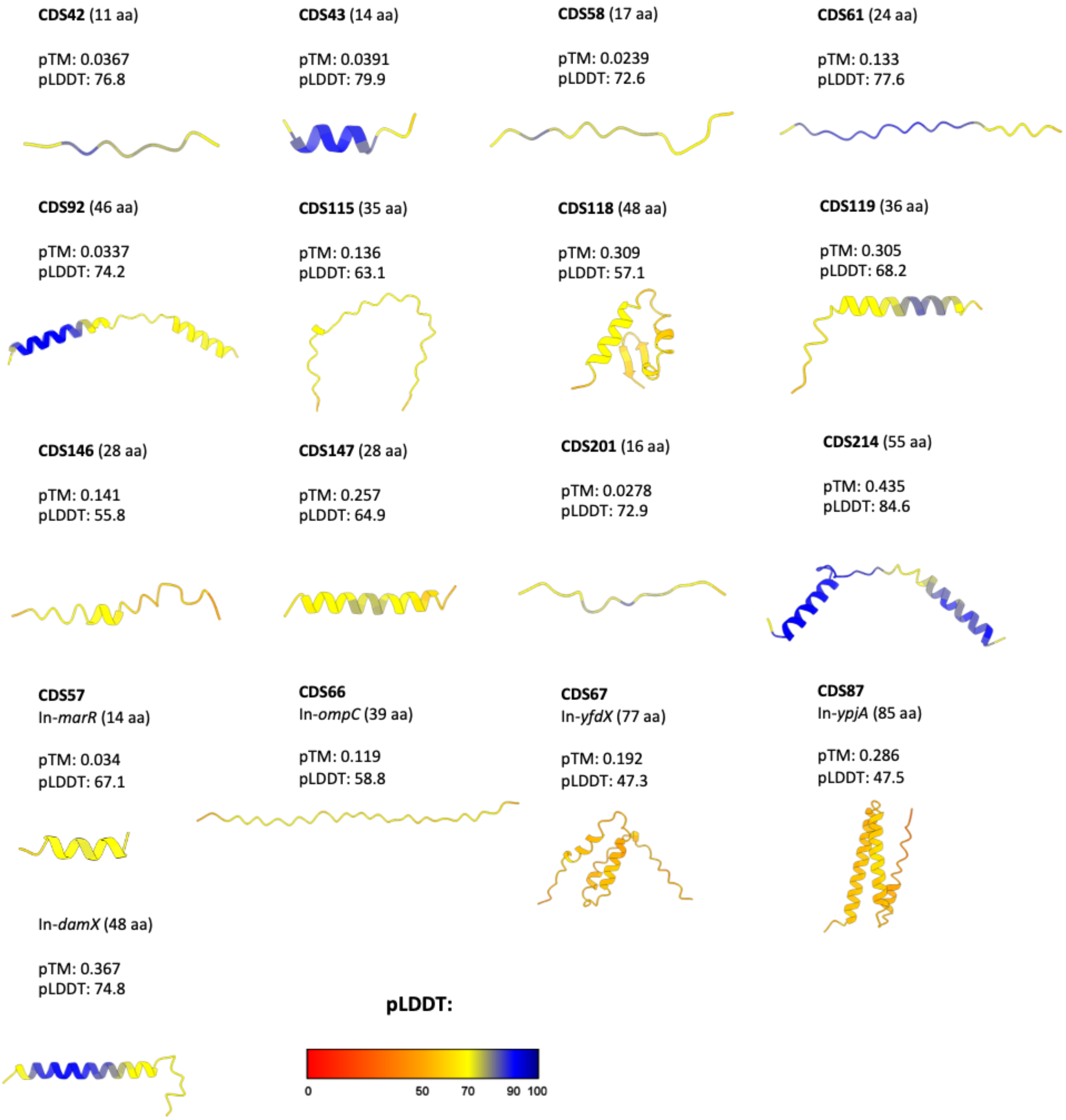

Supplementary Figure 10. Predicted protein structures

Protein structures predicted using AlphaFold (Mirdita et al., 2022). All structures are shown with the N-terminus on the left and coloured by pLDDT using Chimera.

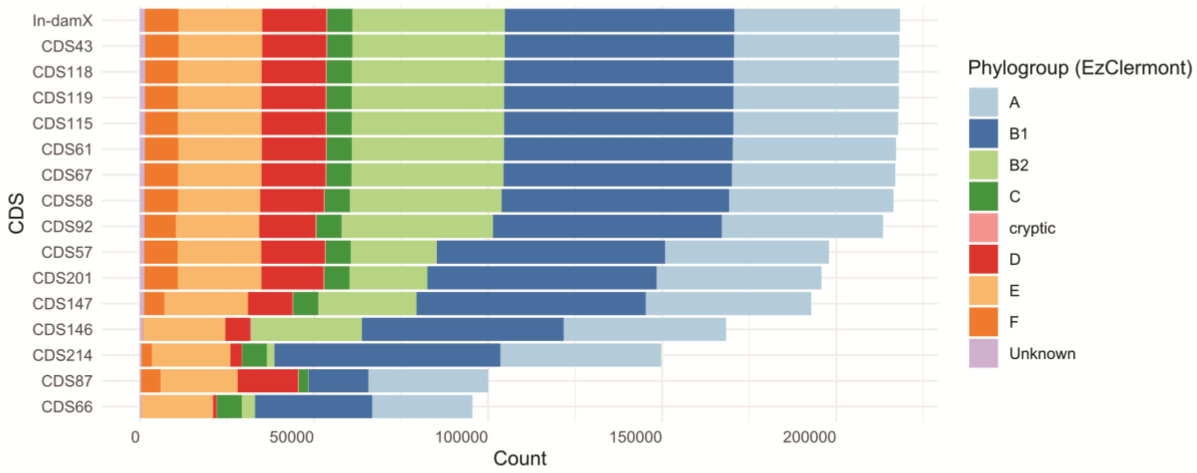

Supplementary Figure 11. Gene conservation within *E. coli*
The phylogenetic distribution of CDS homologs with ≥80% identity and ≥80% coverage.

References

Buchan, D.W.A., Jones, D.T., 2019. The PSIPRED Protein Analysis Workbench: 20 years on. Nucleic Acids Research 47. https://doi.org/10.1093/nar/gkz297

Mirdita, M., Schütze, K., Moriwaki, Y., Heo, L., Ovchinnikov, S., Steinegger, M., 2022. ColabFold: making protein folding accessible to all. Nat Methods 19, 679–682. https://doi.org/10.1038/s41592-022-01488-1
